# Supercritical fluid chromatography-mass spectrometry enables simultaneous measurement of all phosphoinositide regioisomers

**DOI:** 10.1038/s42004-022-00676-6

**Published:** 2022-05-11

**Authors:** Yuta Shimanaka, Keiko Matsumoto, Yuki Tanaka, Yuki Ishino, Shenwei Ni, Jun-Lin Guan, Hiroyuki Arai, Nozomu Kono

**Affiliations:** 1 Department of Health Chemistry, Graduate School of Pharmaceutical Sciences, the University of Tokyo, Tokyo, 113-0033 Japan; 2 Shimadzu Corporation, Kyoto, 604-8511 Japan; 3 Department of Cancer Biology, University of Cincinnati College of Medicine, Cincinnati, OH 45267 USA; 4 Laboratory of Microenvironmental and Metabolic Health Sciences, Center for Disease Biology and Integrative Medicine, Graduate School of Medicine, the University of Tokyo, Tokyo, 113-0033 Japan; 5 AMED-CREST, Japan Agency for Medical Research and Development, Tokyo, 113-0033 Japan

**Keywords:** Lipidomics, Phospholipids

## Abstract

Phosphoinositide species, differing in phosphorylation at hydroxyls of the inositol head group, play roles in various cellular events. Despite the importance of phosphoinositides, simultaneous quantification of individual phosphoinositide species is difficult using conventional methods. Here we developed a supercritical fluid chromatography-mass spectrometry method that can quantify the molecular species of all seven phosphoinositide regioisomers. We used this method to analyze (1) profiles of phosphoinositide species in mouse tissues, (2) the effect of lysophosphatidylinositol acyltransferase 1-depletion on phosphoinositide acyl-chain composition in cultured cells, and (3) the molecular species of phosphatidylinositol-3-phosphate produced during the induction of autophagy. Although further improvement is needed for the absolute quantification of minor phosphoinositide regioisomers in biological samples, our method should clarify the physiological and pathological roles of phosphoinositide regioisomers at the molecular species level.

## Abstract (web summary)

Phosphoinositide (PIP) species play important roles in a number of cellular events, but the simultaneous quantification of individual PIP species remains challenging using conventional methods. Here, the authors report a supercritical fluid chromatography-mass spectrometry approach to simultaneous measure the molecular species of all seven PIP regioisomers.

## Introduction

**Fig. 1:**
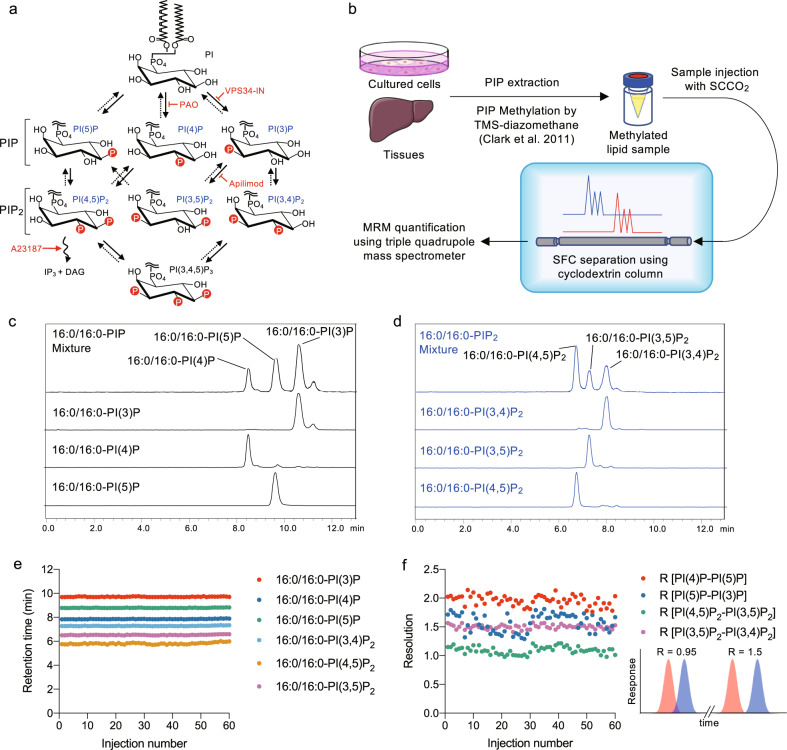
Separation and detection of PIP regioisomers using supercritical fluid chromatography (SFC). **a** Interconversion pathways of seven PIPs. The action of lipid kinases is indicated as solid arrows, and the action of phosphatases is indicated as dashed arrows. The action of PLC is indicated as a wavy arrow. Inhibitors or activators of PIP-metabolizing enzymes were shown in red. **b** A scheme for SFC-MS/MS-based PIP analysis. **c**, **d** Synthetic PIPs were methylated and separated by SFC (see “Methods”). **c** MRM chromatogram (933.6 → 551.6) of a mixture of methylated 16:0/16:0-PI(3)P, 16:0/16:0-PI(4)P, and 16:0/16:0-PI(5)P, or each of them separately. **d** MRM chromatogram (1041.6 → 551.6) of a mixture of methylated 16:0/16:0-PI(3,4)P_2_, 16:0/16:0-PI(3,5)P_2_, and 16:0/16:0-PI(4,5)P_2_, or each of them separately. **e**, **f** Reproducibility of regioisomer separations. A mixture of 16:0/16:0-PI(3)P, -PI(4)P, -PI(5)P, -PI(3,4)P_2_, -PI(3,5)P_2_, and -PI(4,5)P_2_ was methylated and separated by SFC (see Methods) 60 times in succession. **e** Retention time of each 16:0/16:0-PIP regioisomer. **f** Resolution (R) between 16:0/16:0-PIP regioisomers. The calculation of R is described in “Methods”. Two examples of the relationship between resolution and the separation of a two component mixture (blue and red peaks) are also shown in (**f**). Data (**c**, **d**) were collected using an LCMS-8060 mass spectrometer, and (**e**, **f**) were collected using a QTRAP4500 mass spectrometer. Data are from one set of experiments. Images in (**c**, **d**) are representative of three experiments.

Phosphoinositides (PIPs), which are phosphorylated forms of phosphatidylinositol (PI), are minor but essential components of membrane phospholipids. Phosphorylation of phosphatidylinositol (PI) at the 3-, 4-, and 5- hydroxyls of the inositol head group, which is regulated by phosphoinositol kinases and phosphatases, generates seven PIP species in eukaryotes (Fig. 1a). PIPs play crucial roles in cell migration, adhesion, signal transduction, and membrane trafficking. Furthermore, mutations in genes encoding PIP-metabolizing enzymes are associated with the development of diseases, such as cancer and diabetes, as well as psychiatric and neural disorders^1^. Recent studies also suggest the importance of the composition of the two fatty acid chains in PIPs as well as the phosphorylation status of the inositol ring of PIPs in protein functions and the development of cancer^2,3^. However, simultaneous quantification of individual PIP species is challenging because of the technical limitations of conventional methods. Classically, cellular PIPs are measured by labeling cells with [^3^H]inositol and separating deacylated PIP species using high-performance liquid chromatography (HPLC)^4^. However, this method cannot provide information on fatty acyl chains and cannot be applied to tissue samples due to difficulties in radiolabeling. Mass assays based on the phosphorylation of PI(3)P or PI(5)P using recombinant PIKfyve or PIP4K2A in the presence of [^32^P]ATP were also developed to quantify endogenous PI(3)P or PI(5)P in biological samples^5,6^. As an alternative, nonradioactive ELISA-based mass assay kits for PI(3)P, PI(4)P, PI(3,4)P_2_, PI(4,5)P_2_, and PI(3,4,5)P_3_ are commercially available. These assays make it possible to quantify endogenous PIP contents in cell and tissue samples but they also do not provide information on fatty acyl chains. Recently, methylation of the acidic phosphate groups of PIPs has enabled sensitive detection of PIPs by HPLC-electrospray ionization-mass spectrometry (HPLC-ESI-MS) without losing information on fatty acyl chains^7^. However, this method does not distinguish among regioisomers of PIP_1_ and PIP_2_. As shown in Supplementary Fig. 1a, b, this LC-method failed to separate a mixture of methylated PIP_1_ regioisomers [PI(3)P, PI(4)P, and PI(5)P] or a mixture of methylated PIP_2_ regioisomers [PI(3,4)P_2_, PI(3,5)P_2_, and PI(4,5)P_2_]. In this study, we developed a supercritical fluid chromatography (SFC)-ESI-MS/MS (SFC-ESI-MS/MS) method to successfully separate regioisomers of PIP_1_ and PIP_2_ (Fig. 1b).

## Results

### Separation and detection of PIP regioisomers using supercritical fluid chromatography

A supercritical fluid, i.e., a fluid that is maintained above its critical temperature and pressure, has low viscosity and high diffusivity. In supercritical fluid chromatography (SFC), a supercritical fluid is used as the mobile phase and facilitates high-throughput, high-resolution analysis because of the low height equivalent to a theoretical plate (HETP) at high flow rates^8^. We used supercritical carbon dioxide as the mobile phase because it has low polarity and is effective for the analysis of hydrophobic compounds^9,10^. Multiple reaction monitoring (MRM) transitions were selected based on a charged fragment corresponding to diacylglycerol (DAG) (Supplementary Table 1). First, nine stationary phases containing different chemical groups [octadecyl, diol, silica, amine, amide, pyridinyl, poly (4-vinylpyridine), 3-hydroxy phenyl, and β-cyclodextrin groups] were tested under SFC conditions using 0.1% formic acid in methanol/water (97.5/2.5, v/v) as a modifier to separate a mixture of methylated 16:0/16:0-PIP_1_ regioisomers [PI(3)P, PI(4)P, and PI(5)P] or a mixture of methylated 16:0/16:0-PIP_2_ regioisomers [PI(3,4)P_2_, PI(3,5)P_2,_ and PI(4,5)P_2_]. Among the columns tested, only a β-cyclodextrin column successfully separated PIP_1_ and PIP_2_ regioisomers, with sharp peaks and excellent resolution (Fig. 1c, d). On the other hand, the cyclodextrin column failed to separate PIP regioisomers when organic solvents were used as the mobile phase (Supplementary Fig. 1c, d). This method could detect each species of 16:0/16:0-PIPs including PI(3,4,5)P_3_ with femtomole sensitivity (Supplementary Fig. 1e–l). The resolution between two adjacent 16:0/16:0-PIP regioisomers remained above 0.95 even after 60 injections (Fig. 1e, f).

### Measurement of PIP regioisomers in mouse embryonic fibroblasts (MEFs)

Using this methodology, we first analyzed PIPs in immortalized mouse embryonic fibroblasts (MEFs). In MEFs, PI(4)P and PI(4,5)P_2_ were by far the most abundant PIP_1_ and PIP_2_, respectively. Minor PIP_1_s [PI(5)P and PI(3)P] and minor PIP_2_s [PI(3,5)P_2_ and PI(3,4)P_2_] were detected on the shoulders of the PI(4)P and PI(4,5)P_2_ peaks, respectively (Supplementary Figs. 2a, c, 3a, 5a, c, and 6a). The separation became poorer as the length and degree of unsaturation of fatty acyl chains increased, as demonstrated by the 38:4 series. For the measurement of PIP content, we spiked the samples with nonbiological 1-heptadecanoyl-2-arachidonoyl PI(4)P [17:0/20:4-PI(4)P], 17:0/20:4-PI(4,5)P_2_, 17:0/20:4-PI(3,4,5)P_3_, and 12:0/13:0-PI as internal standards. We measured the peak areas of minor PIPs using baselines drawn from valley to valley (Supplementary Figs. 4a–c and 7a–c). The measurements are expressed as PIx/area of the internal standard. PIP_1_ and PIP_2_ regioisomers from 2.5 × 10^4^ ~ 2.5 × 10^5^ cells could be measured with good linearity (Supplementary Figs. 4d and 7d).

The total amount of each PIP regioisomer in biological samples was estimated by summing the amounts of PIP species listed in Supplementary Table 1. For the quantification, we used the calibration curves of 17:0/20:4-PI(3)P, -PI(4)P, -PI(5)P, -PI(3,4)P_2_, -PI(3,5)P_2_, -PI(4,5)P_2_, -PI(3,4,5)P_3_, and 12:0/13:0-PI to estimate the amounts of the corresponding PIPs. This method of quantification is not absolute because it is based on the assumption that the ionization efficiencies of lipid species within each PIP class are the same. The estimated total amounts of PI(3)P, PI(4)P, PI(5)P, PI(3,4)P_2_, PI(3,5)P_2_, and PI(4,5)P_2_ in MEFs were 4.6 ± 0.4, 18.4 ± 1.5, 4.1 ± 0.3, 1.3 ± 0.1, 1.9 ± 0.1, and 20.5 ± 1.0 pmol/10^6^ cells, respectively (Supplementary Table 2). Unexpectedly, the amounts of minor endogenous PIPs [PI(5)P, PI(3)P, PI(3,4)P_2_, and PI(3,5)P_2_] were 3- to 20-fold higher than the amounts measured by the [^3^H]inositol or [^32^P]Pi labeling method^4,11^ (Supplementary Table 2). As shown in Supplementary Fig. 1m, there was no significant difference in methylation efficiency among the synthetic 18:0/20:4-PIP regioisomers.

We then analyzed a synthetic 38:4-PIP_1_ mixture [PI(3)P/PI(4)P/PI(5)P = 0.2/1/0.2] and a synthetic 38:4 PIP_2_ mixture [PI(3,4)P_2_/PI(3,5)P_2_/PI(4,5)P_2_ = 0.1/0.1/1] that mimicked the PIP_1_ and PIP_2_ compositions, respectively, in MEFs, based on our quantitative results (Supplementary Figs. 4e and 7e). As a result of quantifying the synthetic mixtures, the calculated ratios of PI(3)P and PI(5)P to PI(4)P were 0.2 ± 0.004 and 0.2 ± 0.018, respectively, and the calculated ratios of PI(3,4)P_2_ and PI(3,5)P_2_ to PI(4,5)P_2_ were 0.1 ± 0.01 and 0.1 ± 0.03, respectively. These values were comparable to the theoretical values, which demonstrates the quantitative validity of this methodology. To test the possibility that minor PIPs could have been insufficiently labeled in the radioisotope-labeling methods, we also labeled PIPs in MEFs with *d*_*6*_-inositol instead of [^3^H]inositol and quantified them by SFC-MS/MS. The labeling of PIP regioisomers with *d*_*6*_-inositol reached isotopic equilibrium within 72 h (Supplementary Fig. 8a). Labeling efficiency was about the same for 38:4-PIP regioisomers (Supplementary Fig. 8b). However, minor PIPs [PI(5)P, PI(3)P, PI(3,4)P_2_, and PI(3,5)P_2_] were detected at lower levels than they were in other unlabeled experiments: the amount of PI(5)P and PI(3)P was about 14 and 7% of the amount of PI(4)P, respectively, and PI(3,4)P_2_ and PI(3,5)P_2_ was about 2 and 9% of the amount of PI(4,5)P_2_, respectively (Supplementary Fig. 8c). These results suggest that the deviation of the minor PIPs values from those obtained with radioisotope-labeling methods is not due to a low labeling efficiency of minor PIPs, but is partly due to the culture conditions of the labeling experiments. All subsequent measurements are expressed as PIx/internal standard unless otherwise indicated.

To confirm that the peaks identified as regioisomers were not the result of different fatty acyl chain combinations, alternative MRM transitions were programmed for 38:4-PIP_1_, 36:2-PIP_1_, 38:4-PIP_2,_ and 36:2-PIP_2_ (Supplementary Figs. 2b, 3b-e, 5b, and 6b–e). The MRM transitions with daughter ion at m/z 341.1 were selected based on a charged fragment corresponding to 18:0-monoacylglycerol (MAG) (Supplementary Table 1). The chromatograms of 18:0/18:2-PIP_1_, 18:0/20:4-PIP_1_, 18:0/18:2-PIP_2_, and 18:0/20:4-PIP_2_ looked like the chromatograms of 36:2-PIP_1_, 38:4-PIP_1_, 36:2-PIP_2_, and 38:4-PIP_2_, respectively (Supplementary Figs. 2b, 3b, 5b, and 6b, respectively), supporting the idea that the peaks were separated based on the difference in the position of the phosphate group, and not on the combination of fatty acyl chains. As for 36:2-PIP_1_ and 36:2-PIP_2_, additional MRM transitions for possible fatty acyl chain combinations (18:1/18:1-, 16:0/20:2-, and 16:1/20:1-PIPs) were programmed (Supplementary Figs. 3c–e and 6c–e). The signal intensities of 18:1/18:1-PIPs were about half those of 18:0/18:2-PIPs. Neither 16:0/20:2-PIPs nor 16:1/20:1-PIPs were detectable. The retention times of 18:0/18:2-PIP_1_ and 18:1/18:1-PIP_1_ were almost the same and the retention times of 18:0/18:2-PIP_2_ and 18:1/18:1-PIP_2_ were almost the same. These results indicate that 36:2-PIPs detected in MEFs were comprised mostly of 18:0/18:2- and 18:1/18:1-PIPs.

**Fig. 2:**
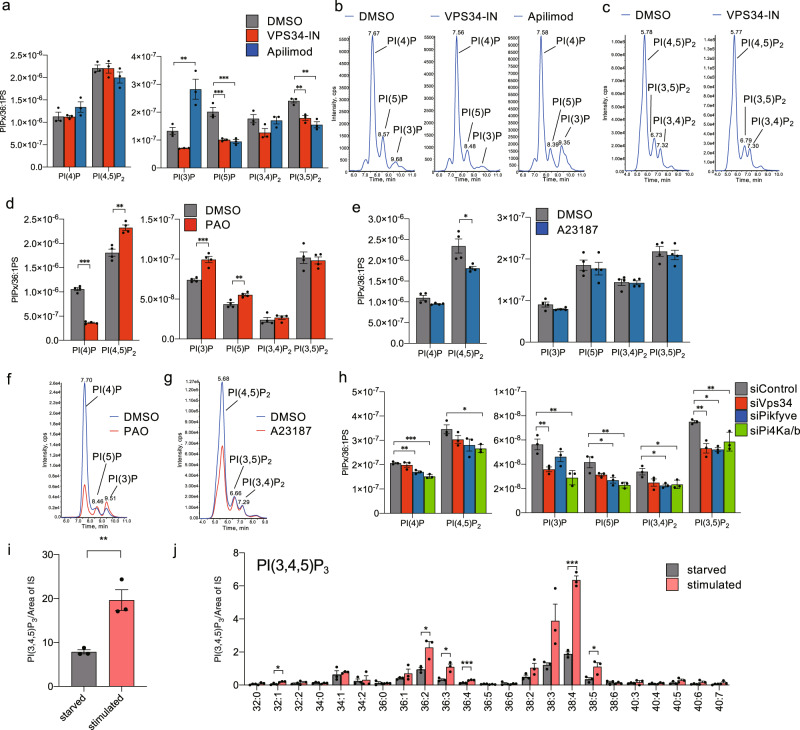
Measurement of PIP regioisomers in immortalized mouse embryonic fibroblasts (MEFs). **a**–**c** MEFs were treated with 1 μM VPS34-IN, 200 nM Apilimod, or vehicle (DMSO) for 90 min, then PIPs were extracted and analyzed using SFC-MS/MS system. **a** The content of individual PIPs in MEFs (1 × 10^6^ cells) treated with 1 μM VPS34-IN, 200 nM Apilimod or vehicle (DMSO) (*n* = 3). **b**, **c** MRM chromatogram [987.6 → 605.6 (**b**) and 1117.6 → 627.6 (**c**)] of extracts from MEFs (1 × 10^6^ cells) treated with VPS34-IN, Apilimod or vehicle (DMSO). **d**–**g** MEFs were treated with 10 μM PAO, 1 μM A23187, or vehicle (DMSO) for 30 min, and then PIPs were extracted and analyzed using SFC-MS/MS system (see “Methods”). **d**, **e** The content of individual PIPs in MEFs (1 × 10^6^ cells) treated with 10 μM PAO, 1 μM A23187 or vehicle (DMSO) (*n* = 4). **f**, **g** Overlay of MRM chromatogram [987.6 → 605.6 (**f**) and 1117.6 → 627.6 (**g**)] of extracts from MEFs (1 × 10^6^ cells) treated with PAO, A23187, or vehicle (DMSO). **h** Contents of individual PIPs in MEFs (1 × 10^6^ cells) transfected with the indicated siRNA (*n* = 3). PIPs were extracted at 48 h after transfection. **i**, **j** Intracellular 38:4-PI(3,4,5)P_3_ content (**i**) and species (**j**) in MEFs. MEF cells cultured in complete medium were serum-starved for 12 h (starved) and then treated with 10% FBS for 10 min (stimulated). PIPs were extracted from MEFs at each step and analyzed using SFC-MS/MS system. Data were collected using a QTRAP4500 mass spectrometer. Values are mean ± s.e.m. Data were analyzed by one-way ANOVA with Dunnett’s test. *P < 0.05, **P < 0.01, and ***P < 0.001 vs vehicle (DMSO). Data are from one set of experiments.

To further validate our methodology, we measured PIPs in MEFs treated with established inhibitors and activators of PIP-metabolizing enzymes (Fig. 1a). Since the inhibitors and activators can affect the cell numbers, PIP contents were also normalized to the endogenous 36:1-PS content, which can be detected simultaneously with PIPs. In mammalian cells, class III PI3-kinase Vps34 produces the majority of PI(3)P and provides most of the pool utilized in the synthesis of PI(3,5)P_2_^12,13^. As expected, treatment with an inhibitor of Vps34 (VPS34-IN^14^), reduced the PI(3)P content to about half of that in vehicle-treated cells (Fig. 2a, b and Supplementary Fig. 9a, b). VPS34-IN also reduced the amount of PI(3,5)P_2_ and PI(3,4)P_2_ (Fig. 2a, c, and Supplementary Fig. 9a, b), suggesting that a large portion of these PIPs is derived from PI(3)P produced by Vps34. VPS34-IN also reduced the PI(5)P content, probably because myotubularin-related proteins (MTMRs) generate PI(5)P by dephosphorylating PI(3,5)P_2_ (Fig. 2a, b, and Supplementary Fig. 9a, b)^11^. VPS34-IN did not affect the amounts of PI(4)P or PI(4,5)P_2_ (Fig. 2a and Supplementary Fig. 9a, b). Apilimod inhibits the activity of type III PIP kinase PIKfyve, which generates PI(3,5)P_2_ from PI(3)P^15^. Apilimod indeed reduced the levels of PI(3,5)P_2_ and PI(5)P, a dephosphorylated product of PI(3,5)P_2_, and increased the level of PI(3)P (Fig. 2a, b, and Supplementary Fig. 9a, b). Inhibition of PI4K activity by phenylarsine oxide (PAO) caused a reduction in cellular PI(4)P content but increased the amounts of PI(4,5)P_2_ (Fig. 2d, f and Supplementary Fig. 9c, d), as previously reported^16^. PAO also slightly increased the amounts of PI(3)P and PI(5)P (Fig. 2d and Supplementary Fig. 9c, d). On the other hand, activation of phospholipase C (PLC) with ionomycin (A23187) caused a reduction in PI(4,5)P_2_ (Fig. 2e, g and Supplementary Fig. 9e, f). We also knocked down the expression of several PIP kinases and quantified PIPs in MEFs (Fig. 2h, Supplementary Fig. 9g, h and Supplementary Fig. 13a–c). *Vps34* suppression decreased PI(3)P, PI(3,4)P_2_, and PI(3,5)P_2_ contents (Fig. 2h), as did *Vps34* knockout (KO) in podocytes^13^. *Pikfyve* suppression decreased PI(5)P and PI(3,5)P_2_ content but did not change the amount of PI(3)P (Fig. 2h), as did the disruption of *Pikfyve* in mouse fibroblasts by gene trapping^11^. PAO inhibits type III PI4-kinases (PI4KA and PI4KB)^17^ and both PI(4)P and PI(4,5)P_2_ contents have been reported to be decreased in *Pi4ka* KO MEFs^18^ and *PI4KB* knocked down COS-7 cells^19^. *Pi4ka* and *Pi4kb* double knockdown decreased the amounts of all PIP species, including PI(4)P and PI(4,5)P_2_ (Fig. 2h). We also measured the cellular PI(3,4,5)P_3_ content in MEFs by our method. As expected^20^, serum stimulation significantly increased the PI(3,4,5)P_3_ content (Fig. 2i, j). Thus, our methodology could identify the whole PIP profiles of the cells.

### Profile of PIP species in mouse tissues

**Fig. 3:**
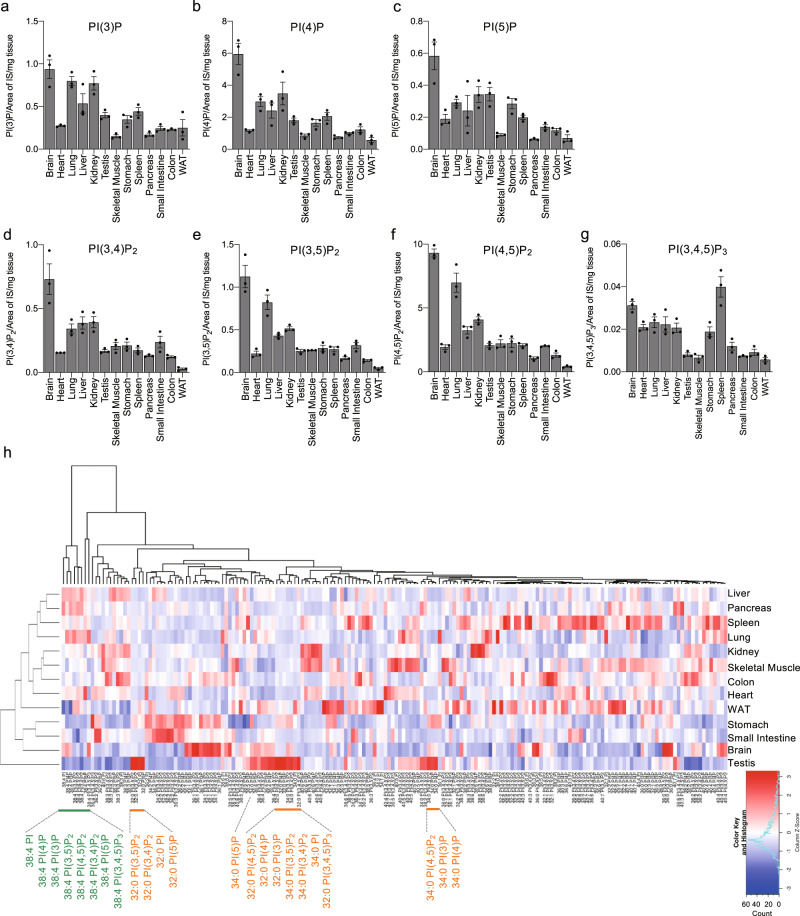
Profile of PIP species in mouse tissues. **a**–**h** PIPs extracted from tissues obtained from 8-week-old male C57BL/6 mice were analyzed by SFC-MS/MS-based method (see “Methods”). **a**–**g** The content of individual PIP classes in the tissues from C57BL/6 mice (*n* = 3). The amounts of PIPs per tissue weight were normalized by the peak area of the corresponding internal standard (IS). **h** Heatmap analysis of the PIP distribution across the 13 mouse tissues. The average amount of each PIP species (*n* = 3) was Z-score normalized across the tissues. The hierarchical clustering of PIP species (columns) and tissues (rows) was based on Pearson’s correlation coefficient to measure the distance and the mean to cluster the samples. The color indicates column Z-score. Values are mean ± s.e.m. *P < 0.05, **P < 0.01, and ***P < 0.001. n.s., not significant. Data are from one set of experiments (**a**–**h**). Data were collected using a QTRAP4500 mass spectrometer.

To gain a global perspective of the distribution of PIPs in mouse tissues, we measured PIP levels across 13 mouse tissues obtained from 8-week-old male mice. As shown in Fig. 3a–g, Supplementary Fig. 10a, and Supplementary Data 1, PIP_1_ and PIP_2_ were more concentrated in the brain, followed by the lung and kidney. PI(3,4,5)P_3_, which is present in very low amounts in unstimulated cells, was concentrated in the spleen. The total PI content in each tissue is shown in Supplementary Fig. 10a. To analyze the characteristics of PIP acyl chain composition in each tissue, 13 tissues were analyzed by a hierarchical clustering analysis based on the amount of PIP species, which was normalized to the total amount of the corresponding PIP classes. As shown in Fig. 3h, hierarchical clustering and heatmap analyses highlighted the differential distribution of PIP molecular species in different tissues. The most distinct cluster of PIP molecular species was comprised of 38:4-PIPs, probably reflecting the fact that 38:4-PIPs are the most dominant and the least variable among the tissues (Fig. 3h, Supplementary Fig. 10b, c, and Supplementary Data 1). On the other hand, the testis differed the most from the other tissues. Interestingly, fully saturated PIP species such as 32:0- and 34:0-PIPs, which were hardly detected in most of the tissues or cultured cells, were abundant in the testis.

### Measurement of the changes in acyl chain compositions of PIP regioisomers

**Fig. 4:**
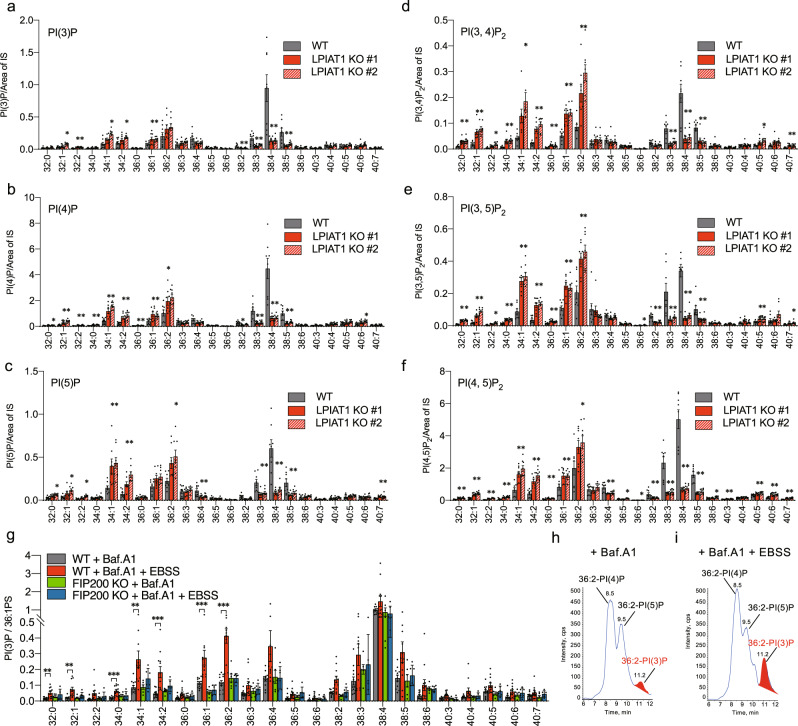
Applications of SFC-MS/MS-based method for analysis of PIPs. **a**–**f** SFC-MS/MS analysis of PI(3)P (**a**), PI(4)P (**b**), PI(5)P (**c**), PI(3,4)P_2_ (**d**), PI(3,5)P_2_ (**e**), and PI(4,5)P_2_ (**f**) species in control and LPIAT1 KO MEFs (*n* = 8). MEFs were transfected with a control or LPIAT1 sgRNA plasmid (#1 or #2). **g**–**i** WT and FIP200 KO MEFs were cultured in full medium or serum-depleted medium (EBSS) in the presence of 100 nM bafilomycin A1 for 30 min. **g** SFC-MS/MS analysis of PI(3)P species in WT MEFs (*n* = 12) and FIP200 KO MEFs (*n* = 6). The variations in total cell lipid input were corrected by endogenous 36:1-PS and levels of PI(3)P species in the indicated conditions are presented with the value of 38:4-PI(3)P content in control (WT + Baf.A1) set as 1. **h**, **i** MRM chromatogram of 36:2-PIP. Methylated lipid extracts from WT MEFs under full medium (**h**) or serum-depleted medium (**i**) in the presence of bafilomycin A1, were separated by SFC and 985.6 → 603.6 MRM transition was monitored. Peaks that increase with serum depletion, which correspond to 36:2-PI(3)P, were highlighted in red. Values are mean ± s.e.m. Data were analyzed by one-way ANOVA with Dunnett’s test. *P < 0.05, **P < 0.01, and ***P < 0.001 vs WT (**a**–**f**) and vs WT + Baf.A1 (**g**). Data are compiled from two (**a**–**f**) or three (**g**) sets of experiments. Images in (**h**, **i**) are representative of three experiments. Data were collected using a QTRAP4500 mass spectrometer.

We next focused on detecting changes in the acyl chain composition of PIPs. PI and PIPs differ from other phospholipids in that most of them have arachidonic acid (20:4n-6) at the *sn-*2 position^21^. This unique feature is formed by lysophosphatidylinositol acyltransferase 1 (LPIAT1) encoded by the *membrane-bound O-acyltransferase domain-containing 7* (*MBOAT7*) gene, which selectively incorporates arachidonic acid into PI^22,23^. We generated LPIAT1 KO MEFs using the CRISPR-Cas9 system with two sgRNAs, #1 and #2 (Supplementary Fig. 11a, b and Supplementary Fig. 13d). As expected^24^, the amount of 20:4-containing PI (38:4) was dramatically reduced in LPIAT1 KO cells (#1 and #2) (Supplementary Fig. 11c). The amount of 20:3-containing PI (38:3), which is often detected in cultured cells^24^, was also reduced in KO cells, while other PI species such as 34:1, 34:2, 36:1, 36:2, 40:5, 40:6, and 40:7 were increased as previously reported^23,24^. Concomitantly with the changes in PI species, 20:4- and 20:3-containing PIPs (38:4 and 38:3) were dramatically reduced by LPIAT1-depletion, while other PIP species such as 34:1, 34:2, 36:1, 36:2, 40:5, 40:6, and 40:7 were increased (Fig. 4a–f). These less unsaturated PIPs (34:1-, 34:2-, 36:1-, and 36:2-PIPs) were probably derived from PI newly produced by PI synthase because mature PI has a 20:4 acyl chain at the *sn*-2 position by the action of LPIAT1^22,23^. The amounts of each PIP regioisomer were unchanged (Supplementary Fig. 11d) regardless of the presence of LPIAT1.

### Less unsaturated PI(3)Ps increase during autophagy induction

Finally, we used this approach to examine the fatty acid composition of PI(3)P produced during autophagosome formation. Macroautophagy (hereafter referred to as autophagy) is a highly conserved catabolic process that involves the delivery of intracellular content to lysosomes for degradation. Upon nutrient deprivation, the Unc-51-like kinase 1 (ULK1) complex [consisting of ULK1, autophagy-related protein 13 (ATG13), RB1-inducible coiled-coil protein 1 (FIP200), and ATG101] triggers nucleation of the phagophore by activating the class III PI3K (PI3KC3) complex I, which in turn activates local PI(3)P production at a characteristic ER structure called the omegasome. Newly produced PI(3)P recruits PI(3)P binding effector proteins such as WD repeat domain phosphoinositide-interacting proteins (WIPIs) and zinc-finger FYVE domain-containing protein 1 (DFCP1). Then, the phagophore membrane expands to form a double-membrane autophagosome, which subsequently fuses with lysosomes for degradation of the engulfed materials^25^. Accumulating evidence suggests that phospholipid synthetic enzymes are recruited to the phagophore and that newly synthesized phospholipids specifically drive phagophore expansion^26,27^. Although the production of PI(3)P by PI3KC3 is a necessary step for autophagosome formation, the molecular species of PI(3)P produced during autophagy induction remain unknown^28,29^. To address this issue, WT MEFs were serum-starved with Earle’s balanced salt solution (EBSS) in the presence or absence of the lysosomal ATPase inhibitor bafilomycin A1 for 30 min and then were subjected to PIP extraction and SFC-ESI-MS/MS analysis. PI(3)P species in FIP200 KO MEFs, which have severe defects in autophagy initiation, were also analyzed as a negative control^30^. Since the EBSS treatment reduced the total lipid content of cells, PIP contents were normalized to the endogenous 36:1-PS content, which is correlated with total lipid contents. After serum starvation, the total amount of PI(3)P in WT MEFs tended to increase in the presence of bafilomycin A1 (Supplementary Fig. 12a). We, therefore, analyzed the fatty acyl species of PI(3)P in the presence of bafilomycin A1. As shown in Fig. 4g, the predominant PI(3)P species was 38:4 in both WT and FIP200 KO MEFs under serum-containing conditions, and serum depletion did not affect the fatty acyl species of PI(3)P in FIP200 KO MEFs. In contrast, serum depletion increased saturated, mono-unsaturated, and di-unsaturated PI(3)P species such as 32:0, 32:1, 34:0, 34:1, 34:2, 36:1, and 36:2, most of which may be derived from newly synthesized PI, in WT MEFs (Fig. 4g–i). No significant changes in other PIP compositions were observed under these conditions (Supplementary Fig. 12b–h). These results suggest that newly synthesized PI is selectively converted to PI(3)P during autophagy induction.

## Discussion

In this study, we developed SFC-ESI-MS/MS-based method that can measure seven PIP regioisomers simultaneously. The most important finding was that PIP regioisomers could be separated by using a supercritical fluid as the mobile phase and an ULTRON AF-HILIC-CD column as the stationary phase. ULTRON AF-HILIC-CD columns possess β-cyclodextrin chemically bonded to a silica stationary phase via a spacer. Since β-cyclodextrin can form an inclusion complex with phospholipids^31^, its ability to separate PIP regioisomers might be due to a difference in the interaction between a β-cyclodextrin group and each PIP regioisomer in the presence of supercritical fluids.

Our SFC-ESI-MS/MS-based method was able to reproducibly separate and robustly quantify the mixture of synthetic PIP regioisomers (Fig. 1c–f, Supplementary Fig. 4e, and Supplementary Fig. 7e). However, in the biological samples, the amounts of minor PIPs [PI(5)P, PI(3)P, PI(3,4)P_2_, and PI(3,5)P_2_] measured by our method were much higher than those measured by radioisotope-labeling methods (Supplementary Table 2). Our *d*_*6*_-inositol-labeling experiments suggest that this discrepancy is partly due to the culture conditions of the labeling experiments. However, it is also possible that the peaks of minor PIPs were inflated by those of the major PIPs. Because PI(4)P and PI(4,5)P_2_ are far more abundant than other PIPs in biological samples, minor PIP_1_s [PI(5)P and PI(3)P] and minor PIP_2_s [PI(3,4)P_2_, and PI(3,5)P_2_] ended up on the shoulders of the large PI(4)P and PI(4,5)P_2_ peaks, respectively. Moreover, a cell-derived extract may cause tailing of the PI(4)P and PI(4,5)P_2_ peaks, which results in inflation of minor shoulder peaks. These effects are greater for PIPs with highly unsaturated fatty acids, which are the predominant molecular species in biological samples because the separation became poorer as the length and degree of unsaturation of fatty acyl chains increased. While our manuscript was under revision, Li and Lämmerhofer reported UHPLC-TripleTOF MS-based method which can measure seven PIP regioisomers with the advantage of chiral chromatography^32^. Notably, their quantification results of endogenous PIP_1_ and PIP_2_ compositions in HeLa cells were in close agreement with our results, except that the PI(3,5)P_2_ concentration is much higher than ours. Further improvements in the separation of PIP regioisomers will resolve the discrepancy in the quantification of minor PIPs between our method and conventional methods.

Nevertheless, our method makes it possible to analyze the changes in the profile of each PIP regioisomer with information on fatty acyl chains in unlabeled biological samples. Using our method, we found that less unsaturated PI(3)P [34:1-, 34:2-, 36:1-, and 36:2-PI(3)P] species were specifically increased during the induction of autophagy in MEFs. These less unsaturated PI(3)Ps were probably derived from PI newly produced by PI synthase because mature PI has a 20:4 acyl chain at the *sn*-2 position by the action of LPIAT1 (Fig. 4). It has been shown that the autophagy-initiating complex is recruited to ER subdomains enriched in PI synthase, and then is translocated to the ATG9A-positive autophagosome precursors in a PI(3)P-dependent manner^27^. A recent in vitro study showed that 34:2-PI is a better substrate for the Vps34 complex than 38:4-PI^33^. These data support the idea that, during autophagosome formation, PI newly synthesized by PI synthase is selectively converted into PI(3)P, which, if true, provides mechanistic insights into how cells rewire PIP metabolism for autophagy regulation.

In conclusion, our SFC-ESI-MS/MS-based method overcomes the technical limitations of the conventional methods for the quantitative measurement of PIPs and makes it possible to analyze and measure fatty acyl species of each PIP regioisomer in murine tissues and cultured cells. However further improvements are needed for the absolute quantification of minor PIPs in biological samples. Our method should lead to a better understanding of the physiological and pathological roles of PIP regioisomers at the molecular species level.

## Methods

### Lipids

12:0/13:0-PI, 17:0/20:4-PI(3)P, 17:0/20:4-PI(4)P, 17:0/20:4-PI(5)P, 17:0/20:4-PI(3,4)P_2_, 17:0/20:4-PI(3,5)P_2_, 17:0/20:4-PI(4,5)P_2_, 17:0/20:4-PI(3,4,5)P_3_ 18:0/20:4-PI(3)P, 18:0/20:4-PI(4)P, 18:0/20:4-PI(5)P, 18:0/20:4-PI(3,4)P_2_, 18:0/20:4-PI(3,5)P_2_ and 18:0/20:4-PI(4,5)P_2_ were from Avanti Polar Lipids. 16:0/16:0-PI(3)P, 16:0/16:0-PI(4)P, 16:0/16:0-PI(5)P, 16:0/16:0-PI(3,4)P_2_, 16:0/16:0-PI(3,5)P_2_, 16:0/16:0-PI(4,5)P_2_, and 16:0/16:0-PI(3,4,5)P_3_ were from Cell Signals.

### Mice

Wild-type mice (C57BL/6J) were purchased from CLEA Japan. All mice were housed in climate-controlled (23 °C) specific pathogen-free facilities with a 12-h light–dark cycle, with free access to standard laboratory food (CE2; CLEA) and water. All animal experiments were performed in accordance with protocols approved by the Animal Committees of the University of Tokyo in accordance with the Standards Relating to the Care and Management of Experimental Animals in Japan.

### Stock solutions for lipid extractions

The following solutions were prepared for lipid extractions; the quench mixture comprising 484 ml MeOH, 242 ml CHCl_3_, and 23.55 ml 1 M HCl; the prederivatization wash composed of 240 ml CHCl_3_, 120 ml MeOH, and 90 ml 0.01 M HCl; and the post-derivatization wash made up of 240 ml CHCl_3_, 120 ml MeOH, and 90 ml H_2_O. Wash mixtures were shaken and allowed to separate into two phases. For pre/post-derivatization washes, the upper phase of each solution was used.

### Cell culture

FIP200 KO MEFs and control MEFs were generated previously^34^. MEFs were maintained in Dulbecco’s modified Eagle’s medium (DMEM; Sigma-Aldrich) supplemented with 10% fetal calf serum, 100 units/ml penicillin, 100 µg ml^−1^ streptomycin, and 2 mM l-glutamine. For treatment with inhibitors or activators of PIP-metabolizing enzymes treatment, 1 × 10^6^ cells were seeded on cell culture dishes (6.0 cm diameter), and the next day, cells were incubated with 1 μM VPS34-IN (Selleck Chemicals) for 90 min, 200 nM apilimod (Selleck Chemicals) for 90 min, 10 μM PAO (Selleck Chemicals) for 30 min, or 1 μM A23187 (Sigma-Aldrich) for 30 min at 37 °C. For autophagy induction, 2 × 10^5^ cells were seeded on six-well plates, and the next day, cells were washed twice with PBS and incubated in EBSS (starvation medium; Thermo Fisher Scientific 24010-043) in the presence or absence of bafilomycin A1 (Selleck Chemicals) for 30 min at 37 °C. After the incubation, cells were washed twice with PBS and collected to a safe-lock poly-propylene tube (2 ml) with 1 M HCl (500 μl), followed by centrifugation (15,000×*g*, 5 min at 4 °C). Supernatants were removed rapidly and resuspended with 750 μl of quench mix, 170 μl of H_2_O, and internal standards [10 μl containing 2 ng 12:0/13:0 PI, 17:0/20:4 PI (4)P, 17:0/20:4 PI (4,5)P_2_, and 17:0/20:4 PI (3,4,5)P_3_], followed by vortex-mixing and lipid extract steps as described below. For knockdown experiments, MEFs were transfected with 5 nM Scramble siRNA (4390843; Thermo Fisher), 5 nM *Vps34* siRNA (Pi3kc3MSS212802; Thermo Fisher), 5 nM *Pykfyve* siRNA (PikfyveMss207628; Thermo Fisher), 5 nM *Pi4ka* siRNA (Pi4kaMSS212530; Thermo Fisher) or 5 nM *Pi4kb* siRNA (Pi4kbMSS200838; Thermo Fisher) using Lipofectamine^TM^ RNAiMAX (Invitrogen) according to the manufacturer’s protocol. For *d*_*6*_-inositol labeling, 7.5 × 10^4^ cells were seeded on 6-cm dishes, and the next day, cells were incubated with myoinositol-depleted Dulbecco’s Modified Eagle’s Medium (Cell Science & Technology Institute) containing 1 or 5 µM myoinositol-*d*_*6*_ (Sigma-Aldrich) and 1 or 5 µM myoinositol (Sigma-Aldrich), respectively. Cells were collected and subjected to PIP analysis after the indicated times.

### Preparation of mouse tissue samples

Isolated mouse tissues were rinsed with cold PBS and immediately frozen in liquid N_2_. Frozen tissues were ground into powder in liquid N_2_ using a pestle and mortar. Frozen tissues were collected into a safe-lock poly-propylene tube (2 ml), and resuspended with 750 μl of quench mix, 170 μl of H_2_O, and internal standards [10 μl containing 2 ng 12:0/13:0 PI, 17:0/20:4 PI (4)P, 17:0/20:4 PI (4,5)P_2_ and 17:0/20:4 PI (3,4,5)P_3_], followed by vortex-mixing and lipid extract steps as described below.

### Lipid extraction from the MEFs and mouse tissues

Lipid extraction was performed, based on procedures described by Clark et al.^7^. The single-phase sample (a mixture of 170 μl of an aqueous sample, 2 ng of internal standards, and 750 μl of quench mix) were mixed with 725 μl of CHCl_3_ and 170 μl of 2 M HCl, followed by vortex-mixing and centrifugation (15,000* × g*, 5 min at room temperature). The lower organic phase was collected into a fresh safe-lock poly-propylene tube (2 ml) and mixed with 708 μl of prederivatization wash, followed by vortex-mixing and centrifugation (15,000* × g*, 3 min at room temperature). The lower phase was collected into another fresh tube and subjected to derivatization, as described below.

### Derivatization of extracted lipids

Derivatization of lipids was performed in a fume hood with the adequate personal safety equipment as follows, based on procedures described by Clark et al.^7^. Fifty microliters of trimethylsilyl diazomethane in hexane (2 M solution; Sigma-Aldrich) was added to the lipid extracts (~1 ml), and after leaving to stand 10 min at room temperature, the reaction was quenched with 6 μl of acetic acid. Next, 700 μl post-derivatization wash solution was added to the mixture, followed by centrifugation (1500 × *g*, 3 min). The resultant lower phase was collected and rewashed with a 700 μl post-derivatization wash solution. Then 90 μl of MeOH and 10 μl of H_2_O were added to the final collected lower phase. The samples were dried up with a centrifugal evaporator. Finally, samples were dissolved in 80 μl MeOH, sonicated briefly, and 20 μl H_2_O was added. To avoid degradation, the samples were stored at −80 °C until use. For an unknown reason, leaving the derivatized PIP samples on the autosampler for more than 24 h (at 4 °C) sometimes resulted in the appearance of a new peak after PI(3)P. In order to obtain reproducible data, the derivatized PIP samples should be analyzed by SFC-ESI-MS/MS within a day of thawing.

### Generation of LPIAT1 knockout MEFs

Single guide RNA (sgRNA) sequences targeting the region adjacent to the exon 3 of *mboat7* were picked using the online CRISPR design tool from the Zhang lab at the Broad Institute. The sgRNA sequences, listed in Supplementary Fig. 2b, were then cloned into the pSpCas9 (BB)-2A-Puro (PX459) vector (a gift from Dr. Feng Zhang, Addgene plasmid # 48139)^35^. These vectors were transfected to MEFs using Lipofectamine 2000 (Invitrogen). Forty-eight hours after transfection, the cells were selected with a fresh medium containing 1 µg ml^−1^ puromycin.

### Western blot analysis

Cells were lysed in SDS-sample buffer [62.5 mM Tris-HCl, pH 6.8, 10% (v/v) glycerol, and 1% (w/v) SDS] with protease inhibitors (0.5 mM phenylmethylsulfonyl fluoride, 2 μg ml^−1^ pepstatin, 2 μg ml^−1^ leupeptin, and 2 μg ml^−1^ aprotinin) and phosphatase inhibitors (5 mM NaF and 2 mM sodium orthovanadate). After sonication, the lysates were used as the total protein extracts. The protein concentrations of samples were determined by the BCA assay (Pierce). Proteins were recovered with an equal volume of 2× SDS-sample buffer [125 mM Tris-HCl, pH 6.8, 20% glycerol, 2% SDS, 200 mM dithiothreitol (DTT) and 0.01% (w/v) bromophenol blue]. Proteins were separated by SDS-PAGE and transferred to PVDF membranes. The membranes were blocked with 5% (w/v) BSA in TTBS buffer [10 mM Tris-HCl, pH 7.4, 150 mM NaCl, and 0.1% (w/v) Tween 20] and incubated with an antibody to GAPDH (6C5; Calbiochem), monoclonal mouse anti-LPIAT1 antibody (clone: FT10)^23^, VPS34 (sc-365404; Santa Cruz), Pikfyve (sc-100408; Santa Cruz), PI4Kα (12411-1-AP; Proteintech), and PI4Kβ (611816; BD laboratory). After incubation with horseradish peroxidase-conjugated anti-mouse or -rabbit antibody (GE Healthcare), the protein was detected by enhanced chemiluminescence (ECL Western blotting detection system, GE Healthcare).

### Total RNA isolation and quantitative real-time PCR

Total RNA was extracted from cells using Isogen II (Nippon-gene) and reverse-transcribed using a High-Capacity cDNA Reverse-Transcriptase Kit (Applied Biosystems). Quantitative real-time PCR was carried out on a LightCycler^®^96 (NIPPON Genetics, Japan) using KAPA SYBR^®^ FAST qPCR Master Mix (NIPPON Genetics, Japan). The sequences of the primers were as follows: Phosphatidylinositol 3-kinase catalytic subunit type 3 (*Vps34*) forward (AGCCCCTGGCCTTACCAGTCAGAA) and reverse (CCGGAAGTTTCAGCCACTCGTTCC); phosphoinositide kinase, FYVE-type zinc-finger containing (*Pikfyve*) forward (ACAGTGCTGAAGAAGGGCTCCCAG) and reverse (TGCGGTTTGTCTGTCCTCCACTGA); phosphatidylinositol 4-kinase alpha (*Pi4ka*) forward (GAAAGCACAGCTCGGAAAGGCAGAG) and reverse (CCCTGCAAGCCACATCAGACAGCA); phosphatidylinositol 4-kinase beta (*Pi4kb*) forward (ACCTGAAACGAACAGCCAGCAACC) and reverse (CAGCCGGACAGGGGAACTGAATGA); actin beta (*Actb*) forward (GCTTCTTTGCAGCTCCTTCGTTGCC) and reverse (CTTTGCACATGCCGGAGCCGTT).

### Electrospray ionization-mass spectrometry (ESI-MS)

For the detection of phospholipids, SFC-MS/MS-based lipidomics analyses were performed on a Shimadzu Nexera UC/s system (Shimadzu) coupled with an LCMS-8060 (Shimadzu) or a QTRAP4500 hybrid triple quadrupole linear ion trap mass spectrometer (AB SCIEX). Lipids extracted were injected by an autosampler; typically, 10 μl of the sample was applied. Chromatographic separation was performed on an ULTRON AF-HILIC-CD (250 mm × 2.1 mm, 5.0 µm; Shinwa Chemical Industries) maintained at 4 °C–10 °C using immersion cooler Neo cool dip BE201F (Yamato Scientific) with mobile phase A [supercritical carbon dioxide (SCCO_2_)] and mobile phase B [water/methanol (2.5/97.5, v/v) containing 0.1% (v/v) formic acid] in a gradient program (0–16 min: 5% B → 20% B; 16.01–18 min: 40% B; 18.01–22 min: 5% B) with a flow rate of 1.5 ml min^−1^. The instrument parameters of LCMS-8060 for positive ion mode were as follows: DL temperature, 250 °C; block heater temperature, 400 °C; interface temperature, 400 °C; nebulizing gas flow, 3 L min^−1^; drying gas flow, 15 L min^−1^; heating gas flow, 5 L min^−1^. The instrument parameters of QTRAP4500 for positive ion mode were as follows: curtain gas, 20 psi; ionspray voltage, 4500 V; temperature, 300 °C; ion source gas 1, 18 psi; ion source gas 2, 20 psi. The specific detection was performed by MRM, as described in Supplementary Table 1.

The resolution (R) between two peaks was calculated as

$${{{{{\rm{R}}}}}}=1.18\times ({t}_{{{{{{\rm{R}}}}}}2}-{t}_{{{{{{\rm{R}}}}}}1})/({W}_{0.5{{{{{\rm{h}}}}}}1}+{W}_{0.5{{{{{\rm{h}}}}}}2})$$

where *t*_R2_ and *t*_R1_ are the retention time of the peaks (*t*_R1_ < *t*_R2_) and *W*_0.5h1_, and *W*_0.5h2_ are the full widths of the peaks at half maximum.

LC-MS/MS analyses of PIPs using an RP column were performed on the Shimadzu Nexera UC/s system coupled with the LCMS-8060 as described previously^7^. Chromatographic separation was performed on an Acquity UPLC C4 BEH column (100 mm × 1 mm, 1.7 μm; Waters) maintained at 40 °C using mobile phase A (water containing 0.1% formate) and mobile phase B (acetonitrile containing 0.1% formate) in a gradient program (0–5 min: 45% B; 5–10 min: 45% B → 100% B; 10–15 min: 100% B; 15–16 min: 100% B → 45% B; 16–20: 45% B) with a flow rate of 0.1 mL min^−1^. The instrument parameters for positive ion mode were as follows: DL temperature, 250 °C; block heater temperature, 400 °C; interface temperature, 300 °C; nebulizing gas flow, 3 L min^−1^; drying gas flow, 10 L min^−1^; heating gas flow, 10 L min^−1^. LC-MS/MS analyses of PIPs using ULTRON AF-HILIC-CD were performed on the Shimadzu Nexera UC/s system coupled with the LCMS-8060. Chromatographic separation was performed on the ULTRON AF-HILIC-CD (250 mm × 2.1 mm, 5.0 µm) maintained at 40 °C using mobile phase A (water containing 0.1% formate) and mobile phase B (methanol containing 0.1% formate) in a gradient program (0–5 min: 45% B; 5–7.5 min: 45% B → 100% B; 7.5–15 min: 100% B; 15.01–20 min: 45% B) with a flow rate of 0.4 mL min^−1^. The instrument parameters for positive ion mode were as follows: DL temperature, 250 °C; block heater temperature, 400 °C; interface temperature, 300 °C; nebulizing gas flow, 3 L min^−1^; drying gas flow, 10 L min^−1^; heating gas flow, 10 L min^−1^.

### Evaluation of methylation efficiency of PIP regioisomers

Methylation efficiency was evaluated by comparing the signals of non-derivatized PIP regioisomers before and after derivatization. Briefly, 1 μg of non-derivatized and derivatized PIP regioisomers, each dissolved in 100 μl MeOH, were prepared. Then, lipids were analyzed by flow injection analysis using a Shimadzu Nexera UC/s system (Shimadzu) coupled with a QTRAP4500 hybrid triple quadrupole linear ion trap mass spectrometer (AB SCIEX) in which the mobile phase was 10 mM ammonium acetate in 50% MeOH in water with 0.2% acetate and the flow rate was 0.2 ml min^−1^. The instrument parameters of QTRAP4500 for negative ion mode were as follows: curtain gas, 30 psi; ionspray voltage, 4500 V; temperature, 700 °C; ion source gas 1, 70 psi; ion source gas 2, 80 psi. The specific detection was performed by MRM, as follows: 38:4 PIP (Q1/Q3, 965.5/321.0; DP, −30; EP, −10; CE, −54; CXP, −11), 38:4 PIP_2_ (Q1/Q3, 1045.5/321.0; DP, −30; EP, −10; CE, −59; CXP, −12).

### Heatmap

The heatmap was created at Shinyheatmap (http://www.shinyheatmap.com) using Euclidean distance metrics and complete linkage algorithms^36^.

### Statistical analysis

Data were converted to means ± s.e.m. values, and the unpaired Student’s *t* test was applied to determine significant differences between the two samples. Statistical differences between multiple treatment groups and a control group were determined using a repeated-measures one-way analysis of variance (ANOVA) with Dunnett’s test. Samples sizes were chosen based on previous experience in our laboratory. The experiments were performed and analyzed in non-randomized and non-blinded fashions. No data were excluded from the analysis. The variance was similar between the groups that were statistically compared.

### Reporting summary

Further information on research design is available in the Nature Research Reporting Summary linked to this article.

## Supplementary information

Description of Additional Supplementary Files

Supplementary Information

Supplementary Data 1

Peer Review File

Reporting Summary

## Data Availability

The datasets generated during and/or analyzed during this study are available in the manuscript, the Supplementary Information, or Supplementary Data 1 and are available from Metabolomics Workbench (Study ID: ST001816, ST001817, and ST001818) and the corresponding author upon request. Source data are provided with this paper.

## Source data

Source Data
