# Supplementary material for: Supercritical fluid chromatography-mass spectrometry enables simultaneous measurement of all phosphoinositide regioisomers
Source: Commun Chem. 2022 May 11;5:61. doi: 10.1038/s42004-022-00676-6 (PMC9814602; doi:10.1038/s42004-022-00676-6)
Supplement: Supplementary file 1 — Description of Additional Supplementary Files [file 42004_2022_676_MOESM1_ESM.pdf]

## Description of Additional Supplementary Files

**File Name:** Supplementary Data 1

**Description:** The estimated content of individual PIP classes and species in the tissues from C57BL/6 mice (n=3).
