# Supplementary material for: Supercritical fluid chromatography-mass spectrometry enables simultaneous measurement of all phosphoinositide regioisomers
Source: Commun Chem. 2022 May 11;5:61. doi: 10.1038/s42004-022-00676-6 (PMC9814602; doi:10.1038/s42004-022-00676-6)
Supplement: Supplementary file 2 — Supplementary Information [file 42004_2022_676_MOESM2_ESM.pdf]

Supplementary Information for

# Supercritical fluid chromatography-mass spectrometry enables simultaneous measurement of all phosphoinositide regioisomers

Yuta Shimanaka[1], Keiko Matsumoto[2], Yuki Tanaka[1], Yuki Ishino[1], Shenwei Ni[1], Jun-Lin Guan[3], Hiroyuki Arai[1,4,5], Nozomu Kono[1,5*]

[1]Department of Health Chemistry, Graduate School of Pharmaceutical Sciences, the University of Tokyo, Tokyo 113-0033, Japan.
[2]Shimadzu Corporation, Kyoto 604-8511, Japan.
[3] Department of Cancer Biology, University of Cincinnati College of Medicine, Cincinnati, Ohio 45267, USA.
[4]Laboratory of Microenvironmental and Metabolic Health Sciences, Center for Disease Biology and Integrative Medicine, Graduate School of Medicine, the University of Tokyo, Tokyo 113-0033, Japan
[5]AMED-CREST, Japan Agency for Medical Research and Development, Tokyo 113-0033, Japan.

*Correspondence: nozomu@mol.f.u-tokyo.ac.jp

# Supplementary Fig. 1

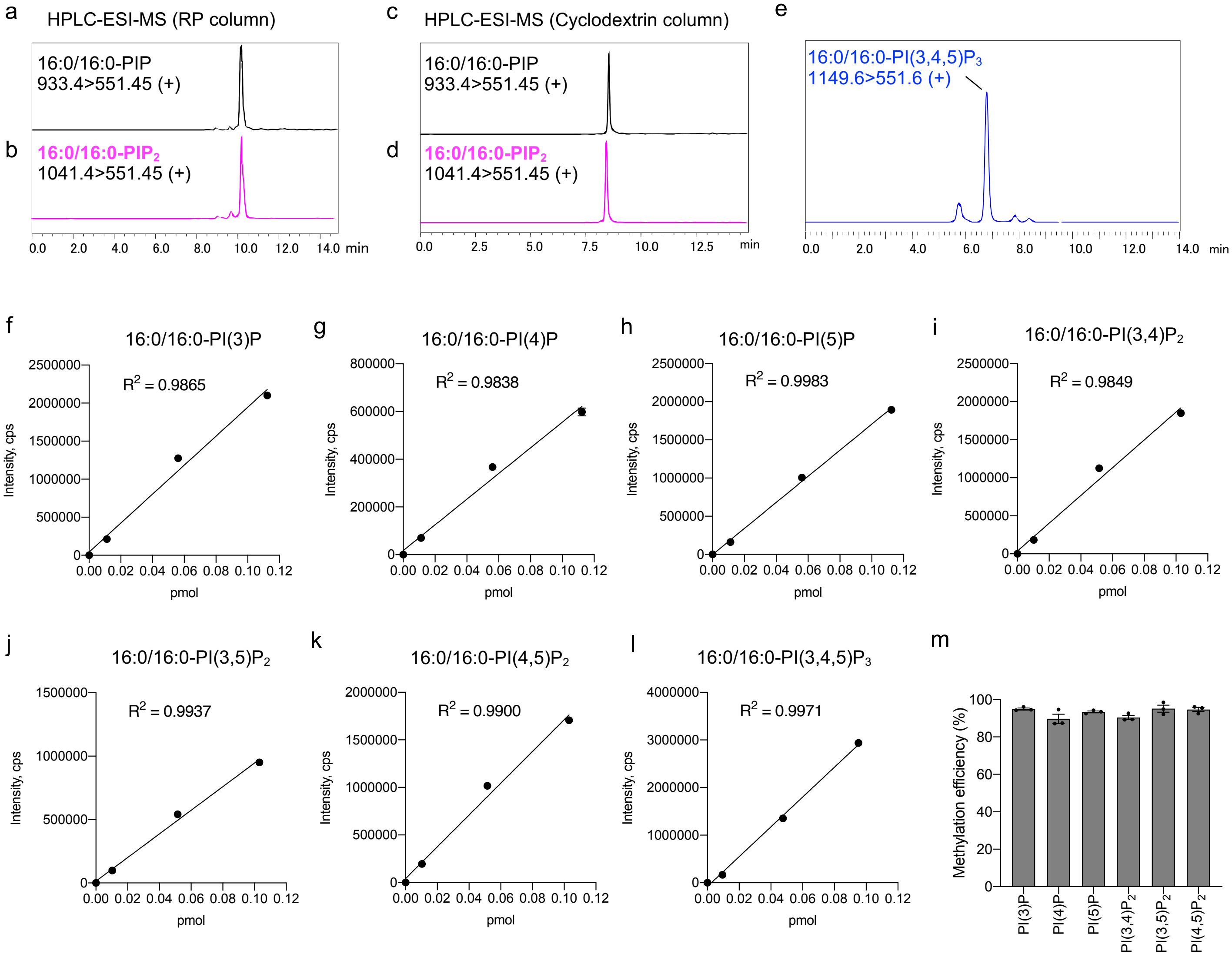


**Supplementary Fig. 1. Validation of supercritical fluid chromatography (SFC)-based method for separating PIP regioisomers.**

(**a, b**) Synthetic PIPs were methylated and separated by the conventional LC method using the RP column (see Methods). MRM chromatogram of a mixture of methylated 16:0/16:0-PI3P, 16:0/16:0-PI4P, and 16:0/16:0-PI5P (**a**), and 16:0/16:0-PI(3,4)$P_2$, 16:0/16:0-PI(3,5)$P_2$, and 16:0/16:0-PI(4,5)$P_2$ (**b**) with parent to daughter transitions as listed on the chromatograms.

(**c, d**) Synthetic PIPs were methylated and separated by the conventional LC method using the cyclodextrin column (see Methods). MRM chromatogram of a mixture of methylated 16:0/16:0-PI3P, 16:0/16:0-PI4P, and 16:0/16:0-PI5P (**c**), and 16:0/16:0-PI(3,4)$P_2$, 16:0/16:0-PI(3,5)$P_2$, and 16:0/16:0-PI(4,5)$P_2$ (**b**) with parent to daughter transitions as listed on the chromatograms.

(**e**) MRM chromatogram (1149.6→551.6) of methylated 16:0/16:0-PI(3,4,5)$P_3$ separated by SFC with the cyclodextrin column (see Methods).

(**f-l**) Calibration curves of synthetic PIP standards of 16:0/16:0-PI(3)P, PI(4)P, PI(5)P, PI(3,4)$P_2$, PI(3,5)$P_2$, PI(4,5)$P_2$, and PI(3,4,5)$P_3$ (n = 3). Diluted series of the methylated PIP standards (10, 50, and 100 pg) were subjected to SFC-MS/MS using the cyclodextrin column (see Methods) and analyzed by MRM.

(**m**) Methylation efficiency of chemically synthesized PIPx 18:0/20:4 (n = 3).

Data are from one set of experiments.

Data (a-l) were collected using a LCMS8060 mass spectrometer and (m) were collected using a QTRAP4500 mass spectrometer.

# Supplementary Fig. 2

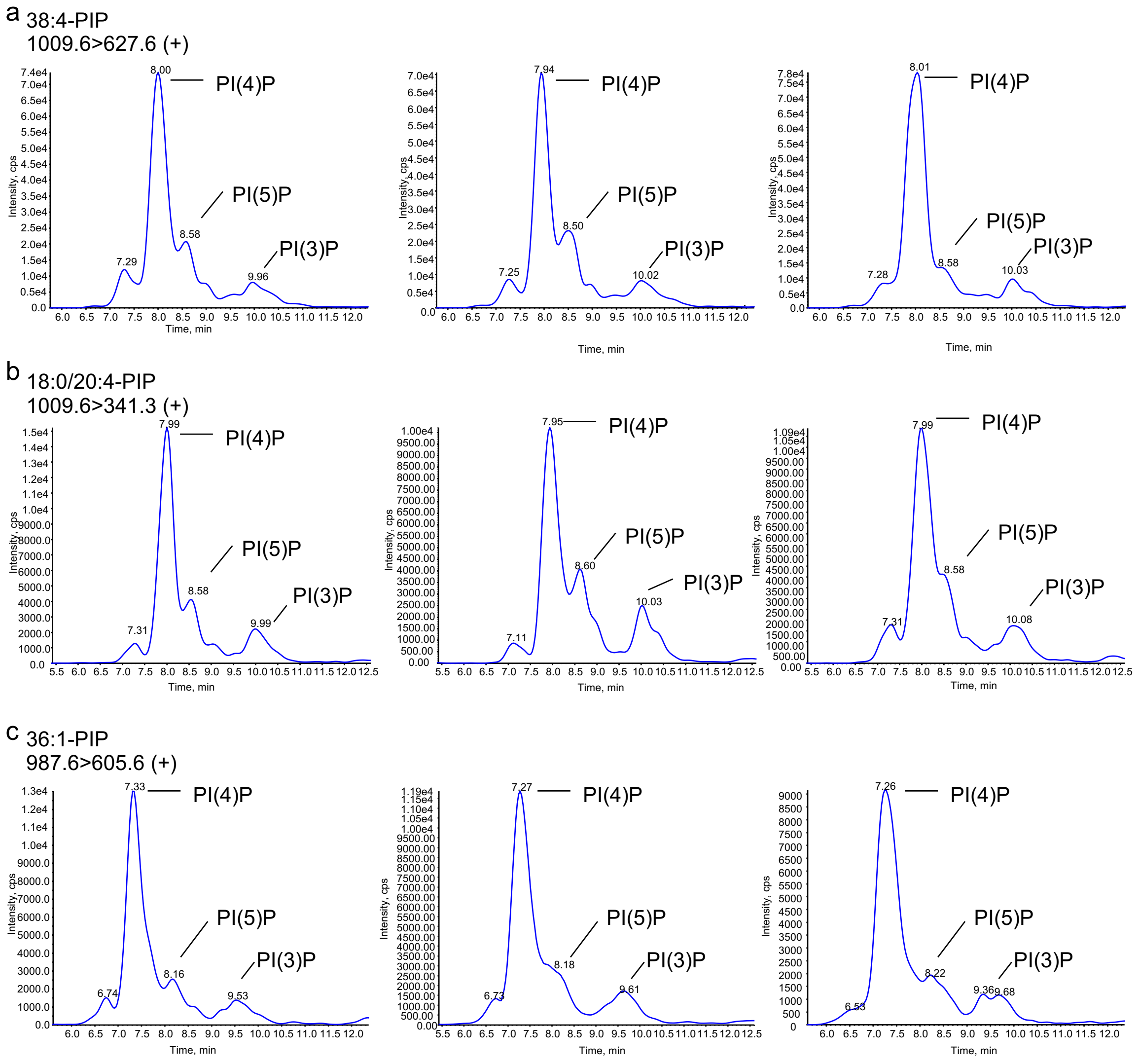


**Supplementary Fig. 2. Analysis of 38:4-PIP and 36:1-PIP in immortalized mouse embryonic fibroblasts (MEFs).**
(**a-c**) PIPs were extracted from MEFs and analyzed using SFC-MS/MS system (see Methods).
(**a**) MRM chromatogram [1009.6→627.6] of methylated lipid extracts from MEFs.
(**b**) MRM chromatogram [1009.6→341.3] of methylated lipid extracts from MEFs.
(**c**) MRM chromatogram [987.6→605.6] of methylated lipid extracts from MEFs.
Three representative chromatograms are shown for each MRM channel. The chromatograms aligned vertically are from the same sample. Data were collected using a QTRAP4500 mass spectrometer.

# Supplementary Fig. 3

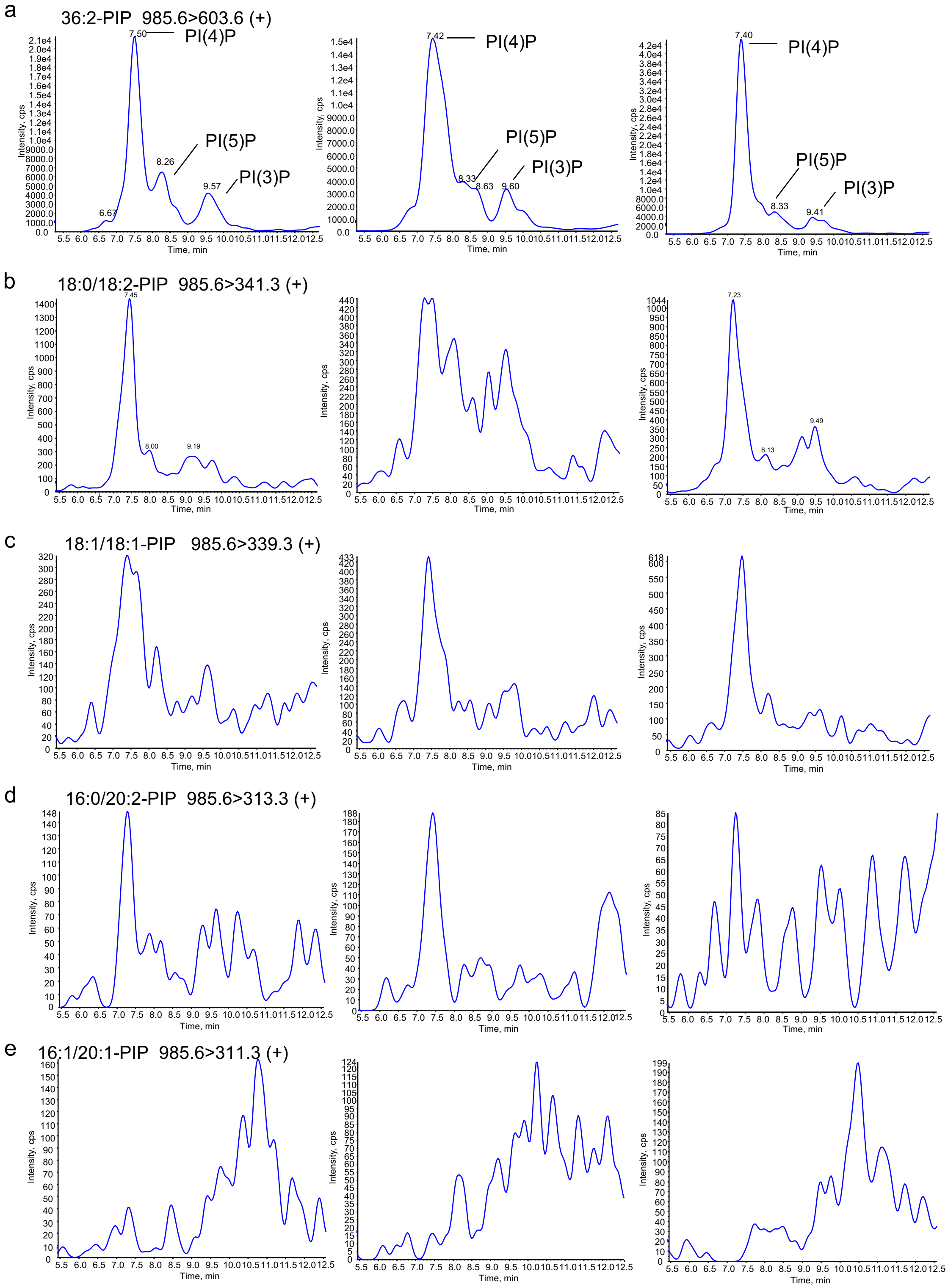


**Supplementary Fig. 3. Analysis of 36:2-PIP from immortalized MEFs.**
(**a-e**) PIPs were extracted from MEFs and analyzed using SFC-MS/MS system (see Methods).
(**a**) MRM chromatogram [985.6→603.6] of methylated lipid extracts from MEFs.
(**b**) MRM chromatogram [985.6→341.3] of methylated lipid extracts from MEFs.
(**c**) MRM chromatogram [985.6→339.3] of methylated lipid extracts from MEFs.
(**d**) MRM chromatogram [985.6→313.3] of methylated lipid extracts from MEFs.
(**e**) MRM chromatogram [985.6→311.3] of methylated lipid extracts from MEFs.
Three representative chromatograms are shown for each MRM channel. The chromatograms aligned vertically are from the same sample. Data were collected using a QTRAP4500 mass spectrometer.

Supplementary Fig. 4

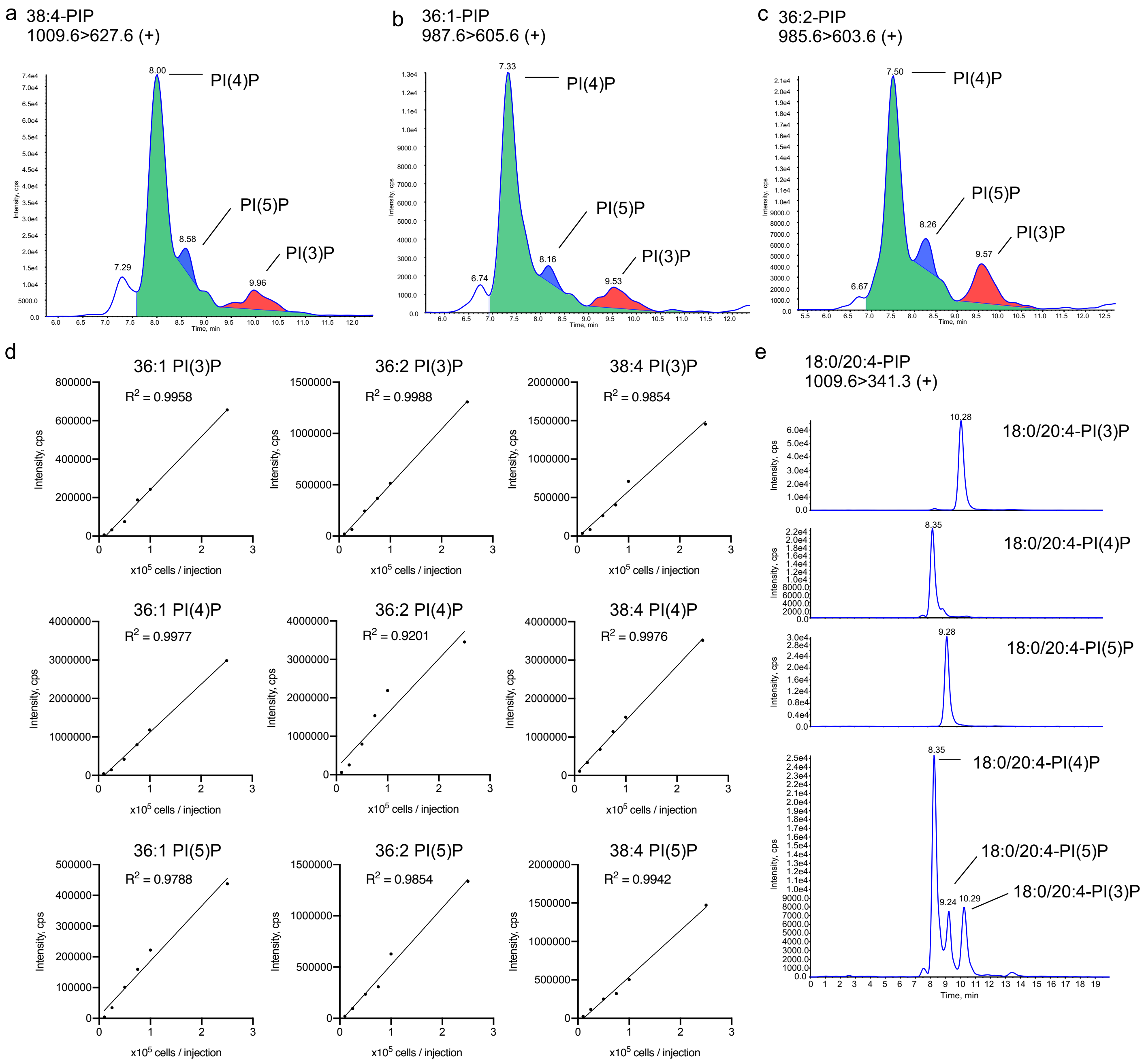


**Supplementary Fig. 4 Quantification of cellular PIP regioisomers separated by SFC.**
(**a-c**) Representative MRM chromatograms of 38:4-PIP (**a**), 36:1-PIP (**b**) and 36:2-PIP (**c**) extracted from MEFs analyzed by SFC-MS/MS system (see Methods). Representative example of the integrations for PI(3)P, PI(4)P and PI(5)P were shown in red, green, and blue, respectively.
(**d**) Relationship between cell number (0.1, 0.25, 0.5, 0.75, 1, 2.5 $\times$ $10^5$ MEFs per injection) and signal intensity of PIP regioisomer (n = 1). The quantification of PIP remained linear up to 2.5 $\times$ $10^5$ cells.
(**e**) MRM chromatograms of methylated 18:0/20:4-PI(3)P, 18:0/20:4-PI(4)P, 18:0/20:4-PI(5)P, and a mixture of methylated 18:0/20:4-PIP regioisomers (PI(3)P/PI(4)P/PI(5)P = 1/5/1).
Data are from one set of experiments. Chromatograms in a-c are the same as Supplementary Fig. 2a, c, and Supplementary Fig. 3a, respectively. Data were collected using a QTRAP4500 mass spectrometer.

# Supplementary Fig. 5

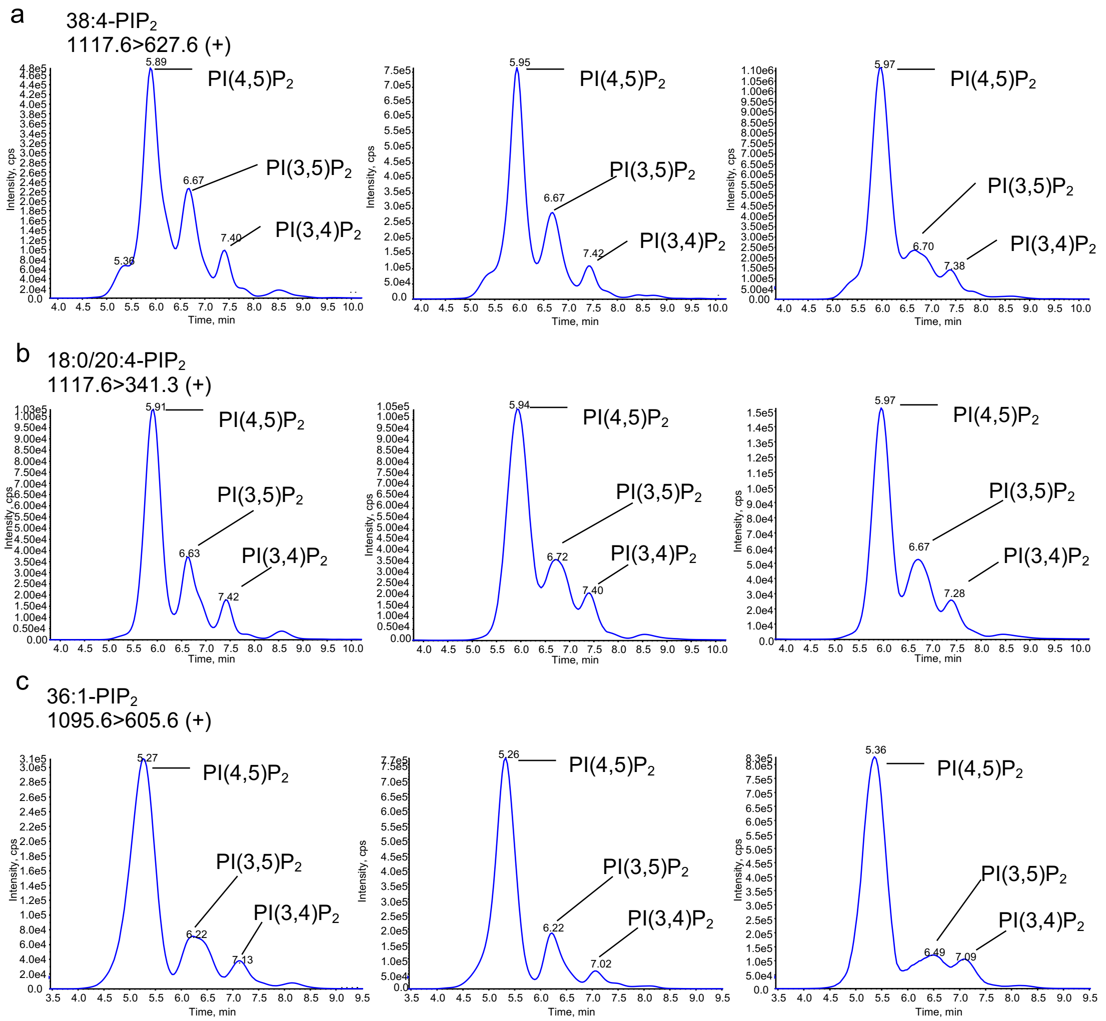


**Supplementary Fig. 5. Analysis of 38:4-PIP$_2$ and 36:1-PIP$_2$ from immortalized MEFs.**
(**a-c**) PIPs were extracted from MEFs and analyzed using SFC-MS/MS system (see Methods).
(**a**) MRM chromatogram [1117.6→627.6] of methylated lipid extracts from MEFs.
(**b**) MRM chromatogram [1117.6→341.3] of methylated lipid extracts from MEFs.
(**c**) MRM chromatogram [1095.6→605.6] of methylated lipid extracts from MEFs.
Three representative chromatograms are shown for each MRM channel. The chromatograms aligned vertically are from the same sample. Data were collected using a QTRAP4500 mass spectrometer.

Supplementary Fig. 6

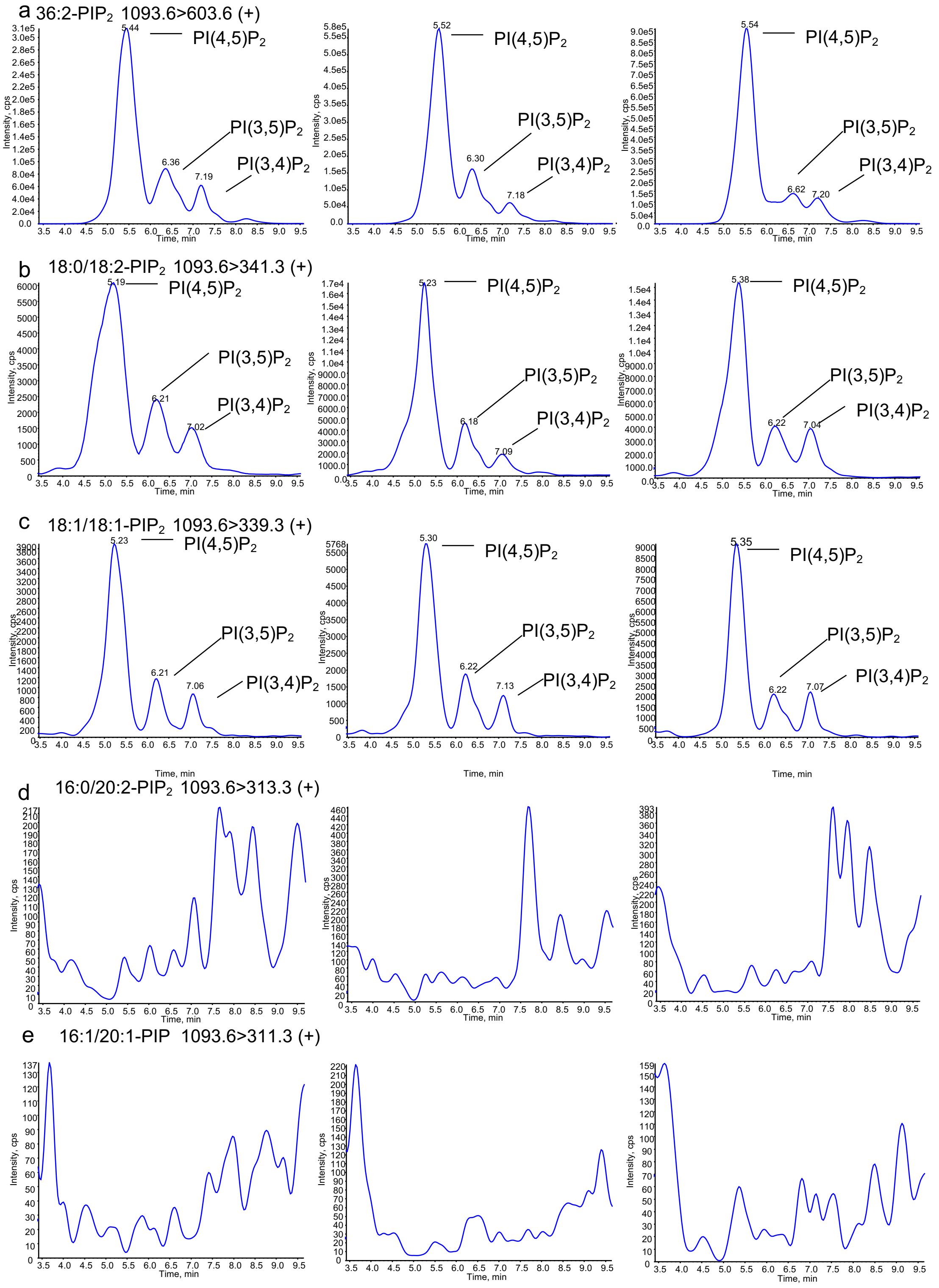


**Supplementary Fig. 6. Analysis of 36:2-PIP$_2$ from immortalized MEFs.**
(**a-e**) PIPs were extracted from MEFs and analyzed using SFC-MS/MS system (see Methods).
(**a**) MRM chromatogram [1093.6→603.6] of methylated lipid extracts from MEFs.
(**b**) MRM chromatogram [1093.6→341.3] of methylated lipid extracts from MEFs.
(**c**) MRM chromatogram [1093.6→339.3] of methylated lipid extracts from MEFs.
(**d**) MRM chromatogram [1093.6→313.3] of methylated lipid extracts from MEFs.
(**e**) MRM chromatogram [1093.6→311.3] of methylated lipid extracts from MEFs.
Three representative chromatograms were shown for each MRM channel. The chromatograms aligned vertically are from the same sample. Data were collected using a QTRAP4500 mass spectrometer.

# Supplementary Fig. 7

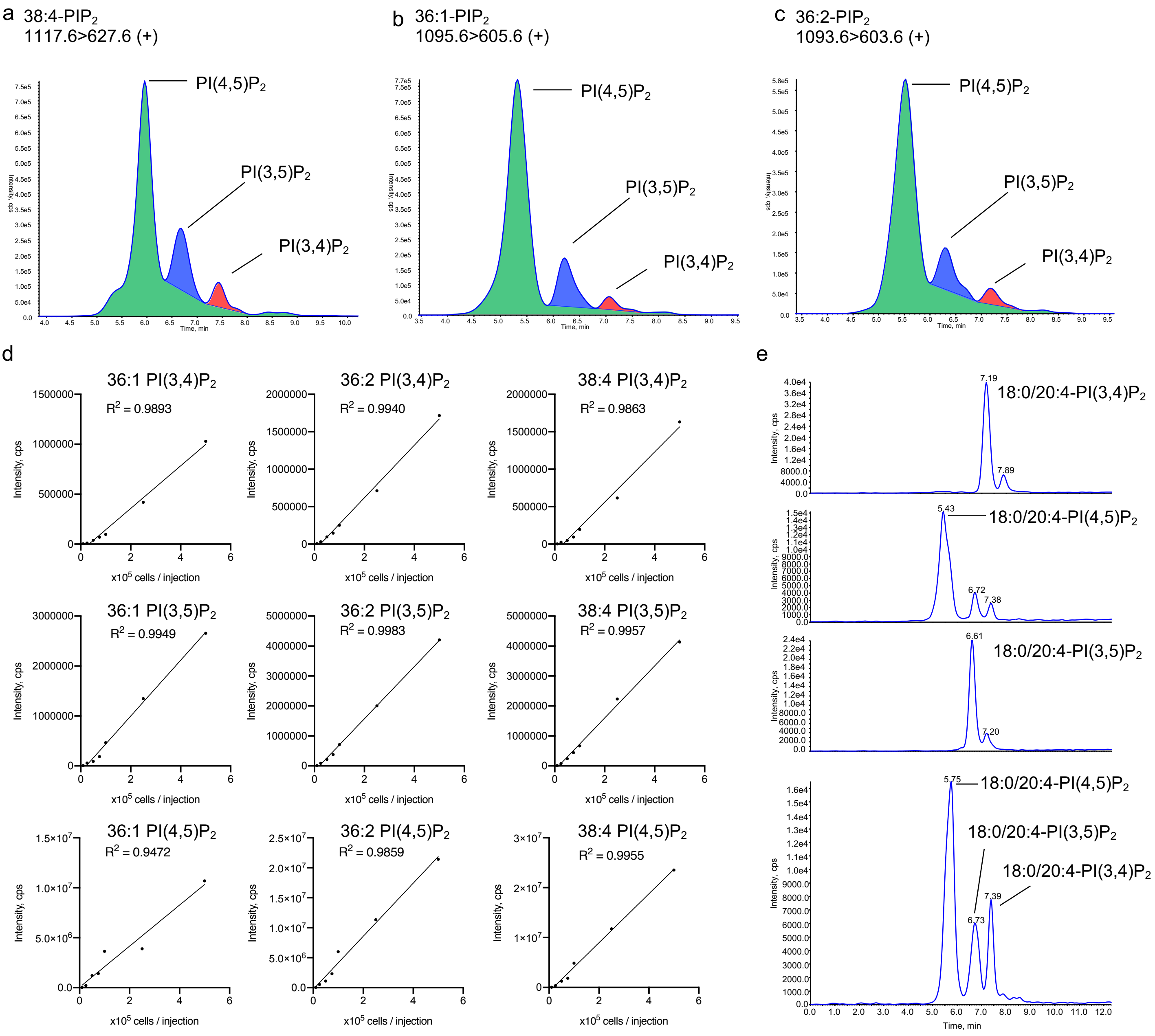


**Supplementary Fig. 7. Quantification of cellular $PIP_2$ regioisomers separated by SFC.**

(**a-c**) Representative MRM chromatograms of 38:4-$PIP_2$ (**a**), 36:1-$PIP_2$ (**b**) and 36:2-$PIP_2$ (**c**) extracted from MEFs analyzed by SFC-MS/MS system (see Methods). Representative example of the integrations for PI(3,4)$P_2$, PI(4,5)$P_2$, and PI(3,5)$P_2$ were shown in red, green and blue, respectively.

(**d**) Relationship between cell number (0.1, 0.25, 0.5, 0.75, 1, 2.5, 5 $\times$ $10^6$ MEFs per injection) and detected $PIP_2$ regioisomer (n = 1). The quantification of $PIP_2$ remained linear up to 5 $\times$ $10^5$ cells.

(**e**) MRM chromatograms of methylated 18:0/20:4-PI(3,4)$P_2$, 18:0/20:4-PI(3,5)$P_2$, 18:0/20:4-PI(4,5)$P_2$, and a mixture of methylated 18:0/20:4-$PIP_2$ regioisomers [PI (3,4)$P_2$ /PI(3,5)$P_2$ /PI (4,5)$P_2$ = 1/1/10].

Data are from one set of experiments. chromatograms in a-c are the same as Supplementary Fig. 5a, c, and Supplementary Fig. 6a. Data were collected using a QTRAP4500 mass spectrometer.

# Supplementary Fig. 8

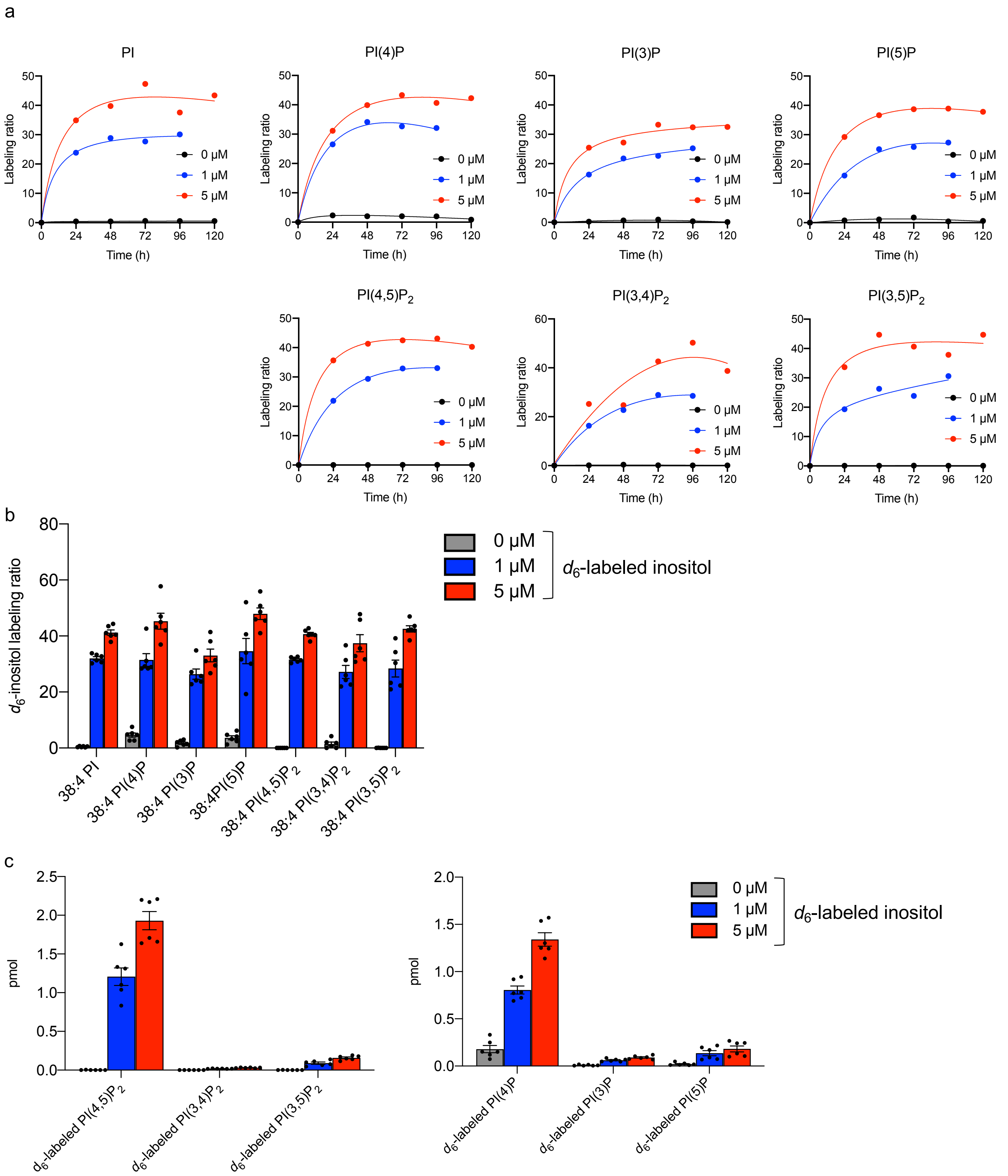


**Supplementary Fig. 8. Quantification of $d_6$-inositol-labeled PIP regioisomers by SFC-MS/MS system.**
MEF cells were cultured in myo-inositol-depleted Dulbecco's Modified Eagle's Medium containing 1, or 5 μM $d_6$-inositol and 1, or 5 μM myo-inositol, respectively. PIPs were extracted from MEFs and analyzed using SFC-MS/MS system (see Methods).
(**a**) $d_6$-inositol-labeling ratio of individual PIP regioisomers after $d_6$-inositol treatment for indicated times (n = 1).
(**b, c**) $d_6$-inositol-labeling ratio of 38:4-series PIP regioisomers (**b**) and estimated content of individual $d_6$-inositol-labed PIP regioisomers (**c**) in MEF cells after 72-hour-$d_6$-inositol treatment (n = 6).
Data are from one set of experiments (a) and compiled from two (b, c) sets of experiments. Data were collected using a QTRAP4500 mass spectrometer.

Supplementary Fig. 9

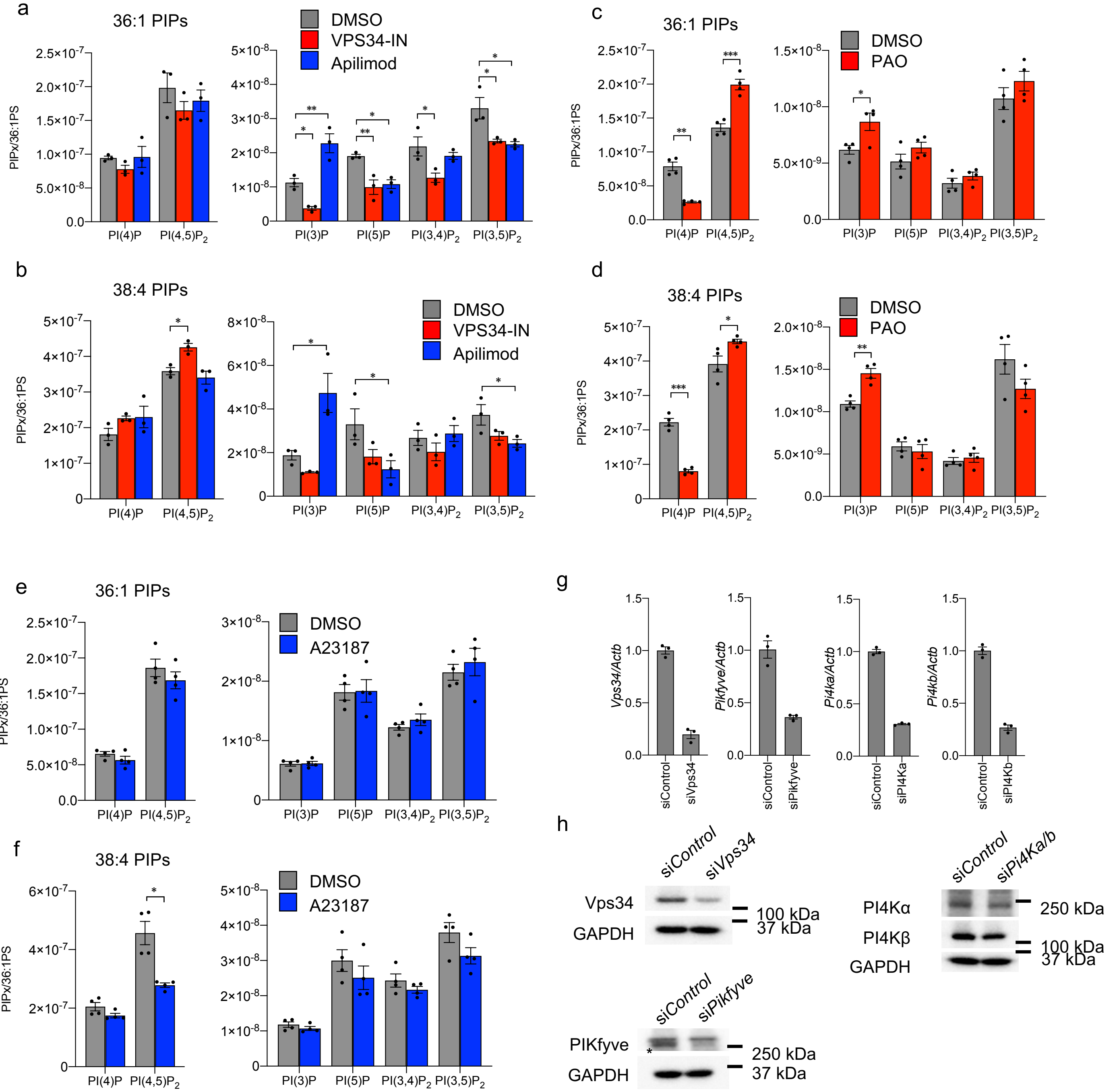


**Supplementary Fig. 9. Changes in PIP profiles from immortalized MEFs upon inhibitions of PIP synthesis or stimulation of PIP degradation.**
(**a, b**) MEFs (1 × $10^6$ cells) were treated with 1 μM VPS34-IN, 200 nM Apilimod, or vehicle (DMSO) for 90 min, then PIPs were extracted and analyzed using SFC-MS/MS system (see Methods).
(**a**) Content of 36:1 PIP regioisomers (n = 3).
(**b**) Content of 38:4 PIP regioisomers (n = 3).
(**c-f**) MEFs (1 × $10^6$ cells) were treated with 10 μM PAO, 1 μM A23187, or vehicle (DMSO) for 30 min, and then PIPs were extracted and analyzed using SFC-MS/MS system.
(**c, e**) Content of individual 36:1 PIP regioisomers (n = 4).
(**d, f**) Content of individual 38:4 PIP regioisomers (n = 4).
(**g, h**) Knockdown efficiency of PIP kinases in MEFs was validated by qPCR (g) and western blotting (h).
Values are mean ± s.e.m. Data were analyzed by one-way ANOVA with Dunnett's test. *$p$<0.05, **$p$<0.01, and ***$p$<0.001vs vehicle (DMSO).
Data (**a-g**) and images in e are from one experiments. Data were collected using a QTRAP4500 mass spectrometer.

# Supplementary Fig. 10

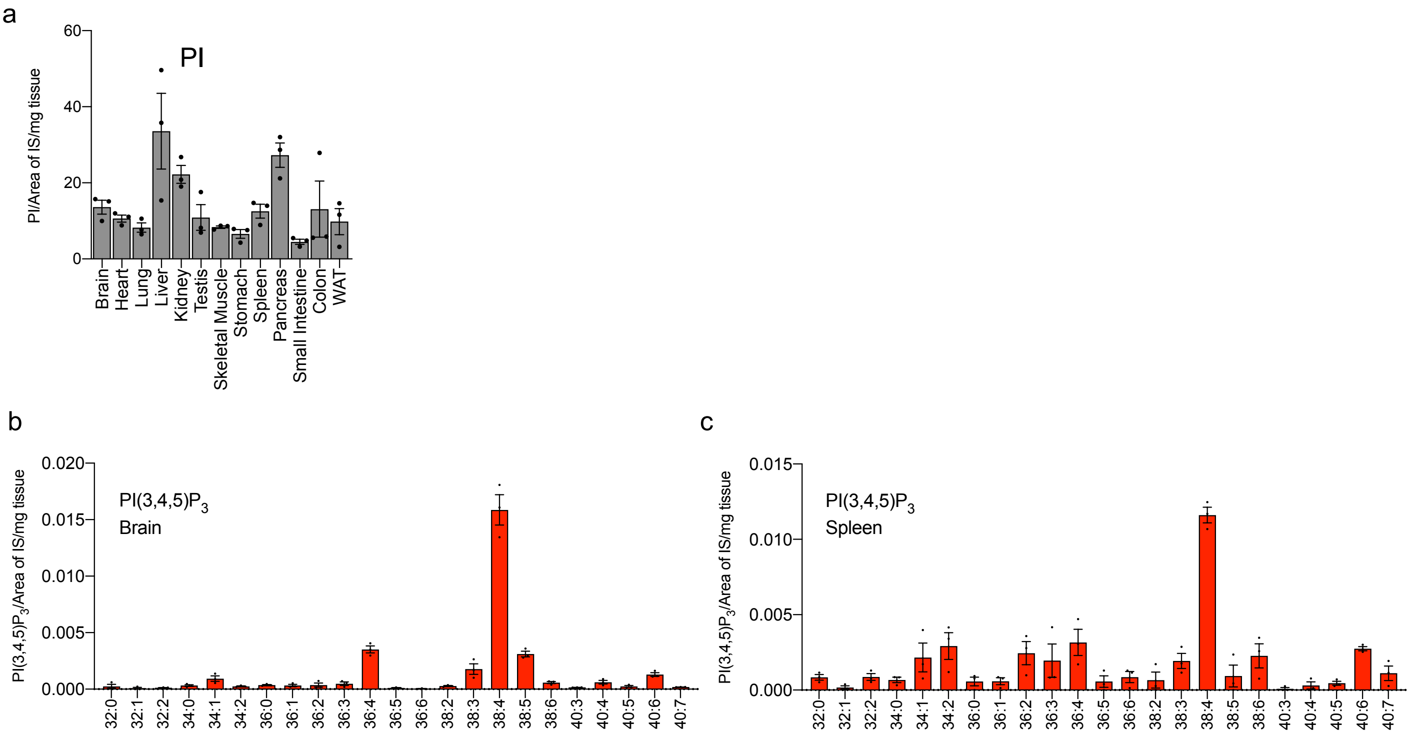


**Supplementary Fig. 10. Levels of PIP regioisomers in mouse tissues**
(**a**) The PI content in the tissues from C57BL/6 mice (n = 3).
(**b, c**) SFC-MS/MS analysis of PI(3,4,5)$P_3$ species in mouse brain (h) and spleen (i) (n = 3).
The amounts of PIPs per tissue weight was normalized by the peak area of the corresponding internal standard (IS). Values are mean ± s.e.m. Data are from one set of experiment. Data were collected using a QTRAP4500 mass spectrometer.

Supplementary Fig. 11

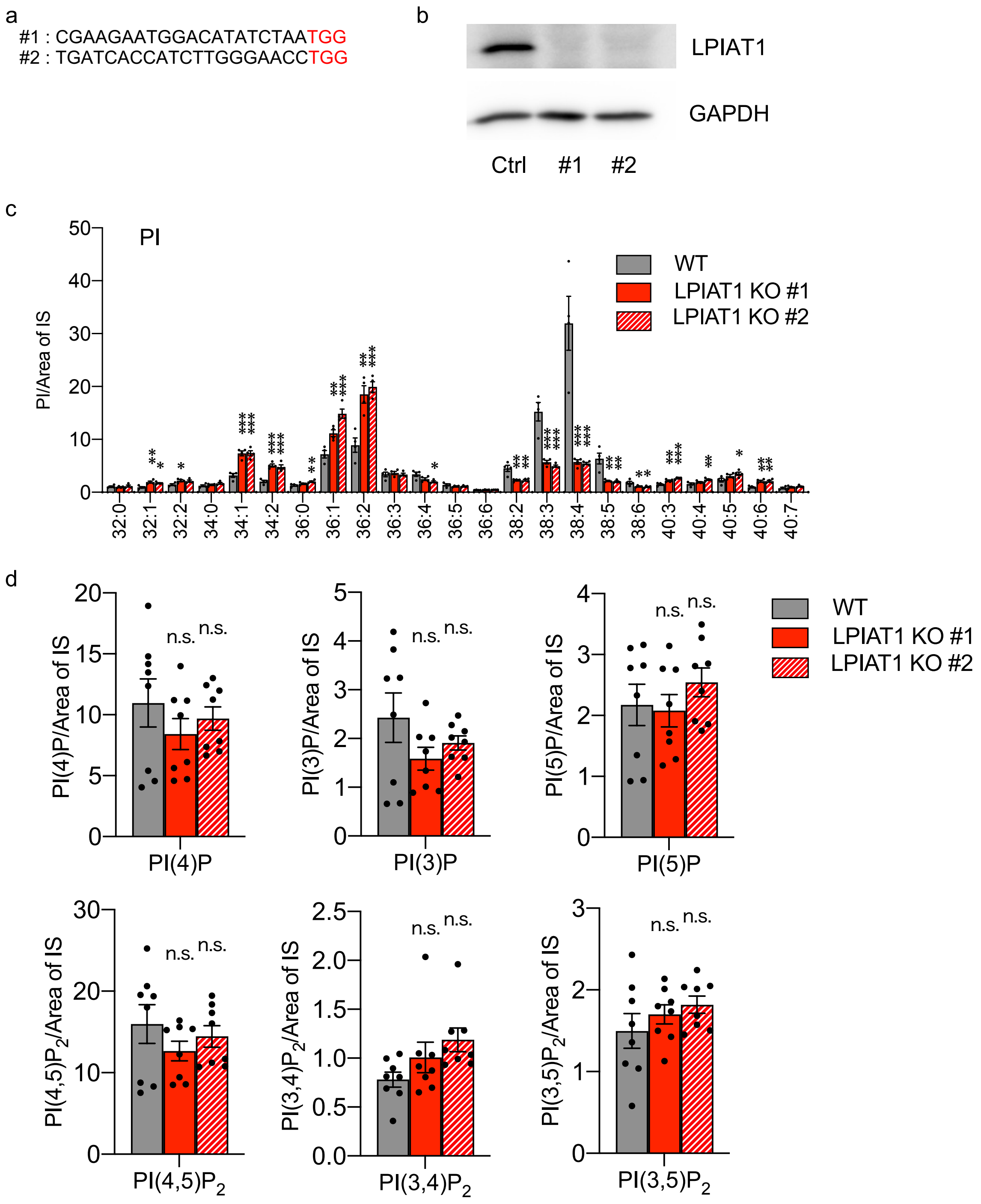


**Supplementary Fig. 11. Analysis of PIP regioisomers in LPIAT1 KO MEFs.**
(**a-d**) MEFs were transfected with a control or LPIAT1 sgRNA plasmid (#1 or #2), and PIP content was analyzed.
(**a**) The sgRNA sequences used to generate LPIAT1 KO MEFs (#1 and #2).
(**b**) Western blot of LPIAT1 in control and LPIAT1 KO MEFs (#1 and #2). GAPDH serves as a control.
(**c**) SFC-MS/MS analysis of PI species in control and LPIAT1 KO MEFs (n = 4).
(**d**) The content of individual PIP classes in control and LPIAT1 KO MEFs (n=8).
Values are mean ± s.e.m. Data were analyzed by one-way ANOVA with Dunnett's test. *$p$<0.05, **$p$<0.01, and ***$p$<0.001, n.s., not significant vs WT.
Data are from one set of experiments (c) and compiled from three (d) sets of experiments. Images in b are representative of one experiment. Data were collected using a QTRAP4500 mass spectrometer.

# Supplementary Fig. 12

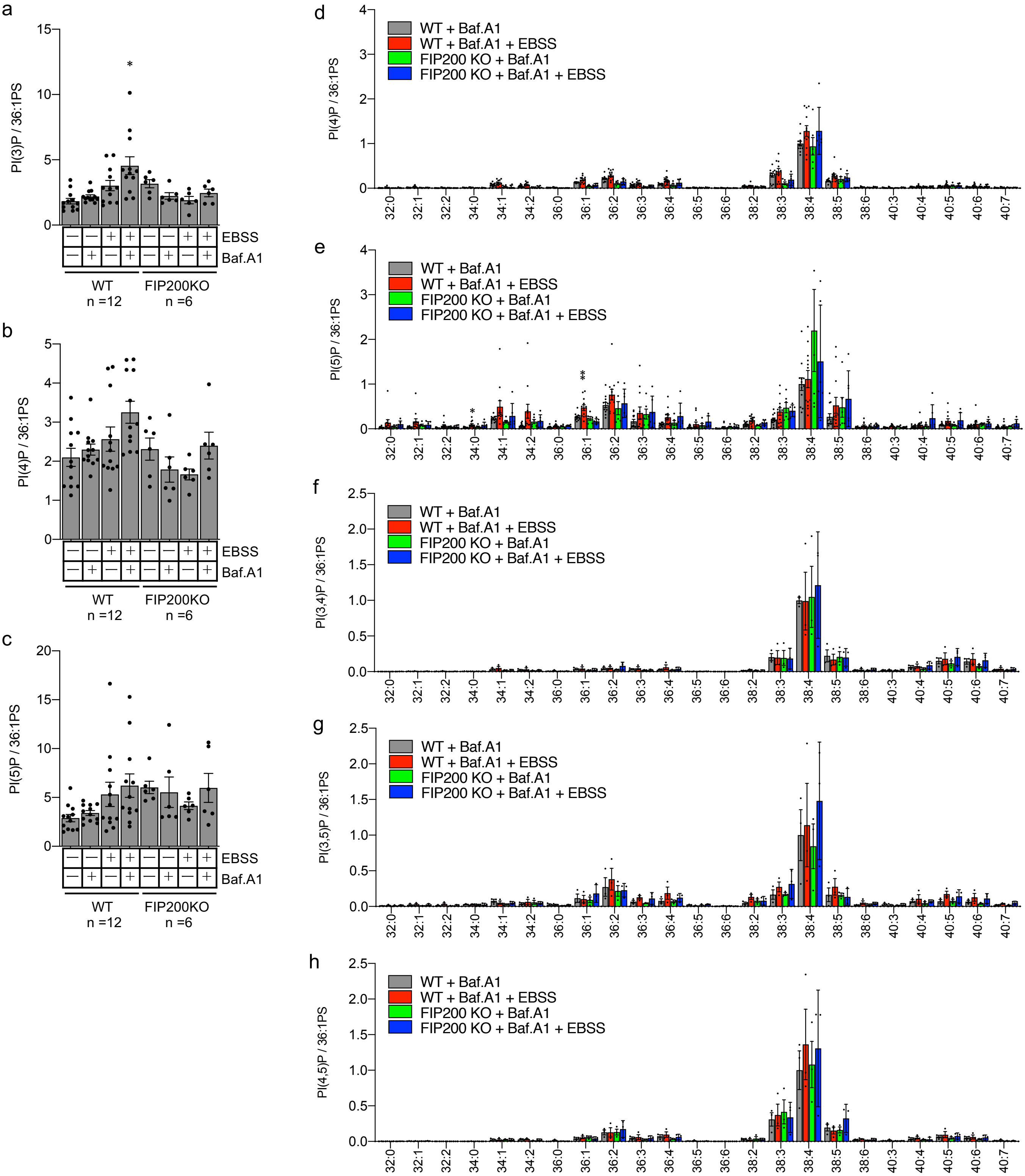


**Supplementary Fig. 12**. **Analysis of PIP regioisomers during autophagy induction.**
WT and FIP200 KO MEFs were cultured in full medium or serum-depleted medium in the presence of or absence of 100 nM bafilomycin A1 for 30 min.
(**a-c**) Total contents of PI (3)P (a), PI(4)P (b), and PI(5)P (c) in WT or FIP200 KO MEFs cultured in full medium or serum-depleted medium in the presence or absence of 100nM bafilomycin A1 for 30 min.
(**d-h**) PIP species in WT MEFs (n = 12) and FIP200 KO MEFs (n = 6). The variations in total cell lipid input were corrected by endogenous 36:1-PS. The levels of PIP species in the indicated conditions are presented with the value of 38:4-PIP content in control (WT + Baf.A1) set as 1.
Values are mean ± s.e.m. Data were analyzed by one-way ANOVA with Dunnett's test. **p*<0.05. n.s., not significant vs WT + Baf.A1.
Data are from one set of experiments (f, g, h) and compiled from three (a, b, c) sets of experiments. Data were collected using a QTRAP4500 mass spectrometer.

Supplementary Fig. 13

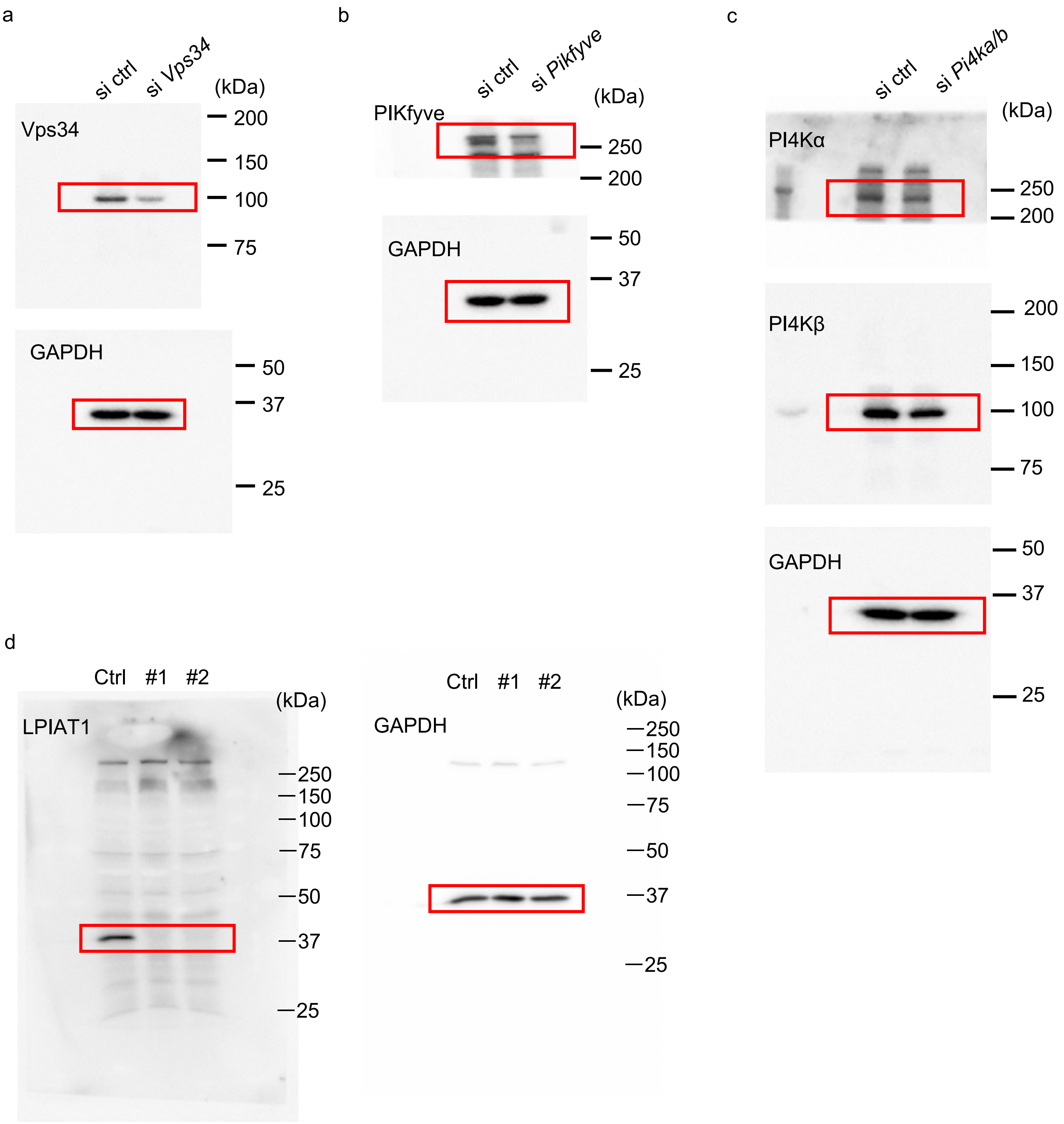


**Supplementary Fig. 13. Original western blotting images used in this study.**
(**a-c**) Full western blotting images used in Supplementary Fig. 9h.
(**d**) Full western blotting images used in Supplementary Fig. 11b.

# Supplementary Tables

**Supplementary Table 1**. Optimized MRM pairs and parameters of mass spectrometer for PIPs.

QTRAP 4500 mass spectrometer (AB SCIEX) parameters

| PIPs Class | DP | EP | CE | CXP |
|---|---|---|---|---|
| PIP | 130 | 10 | 33 | 13 |
| PIP2 | 150 | 10 | 37 | 17 |
| PIP3 | 150 | 10 | 39 | 15 |
| PI | 100 | 10 | 30 | 15 |

LCMS-8060 mass spectrometer (Shimadzu) parameters

| PIPs Class | Target Q1 Pre Bias | Target Collision Energy | Target Q3 Pre Bias |
|---|---|---|---|
| PIP | 24 | 23 | 20 |
| PIP2 | 30 | 30 | 20 |
| PIP3 | 32 | 31 | 20 |

| | Fatty acyl species | Q1 Mass (Da) | Q3 Mass (Da) | Retention Time (min) | | |
|---|---|---|---|---|---|---|
| | | | | PI(3)P | PI(4)P | PI(5)P |
| PIP | 32:0 | 933.6 | 551.6 | 11.7 | 9.1 | 10.0 |
| | 32:1 | 931.6 | 549.6 | 11.6 | 9.3 | 10.1 |
| | 32:2 | 929.6 | 547.6 | 12.0 | 9.5 | 10.5 |
| | 34:0 | 961.6 | 579.6 | 11.8 | 8.8 | 10.0 |
| | 34:1 | 959.6 | 577.6 | 11.7 | 9.5 | 10.2 |
| | 34:2 | 957.6 | 575.6 | 13.2 | 10.6 | 11.2 |
| | 36:0 | 989.6 | 607.6 | 13.4 | 8.6 | 10.0 |
| | 36:1 | 987.6 | 605.6 | 13.1 | 10.2 | 10.1 |
| | 36:2 | 985.6 | 603.6 | 13.2 | 10.4 | 10.3 |
| | 36:3 | 983.6 | 601.6 | 13.7 | 6.5 | 11.7 |
| | 36:4 | 981.6 | 599.6 | 13.6 | 10.8 | 11.6 |
| | 36:5 | 979.6 | 597.6 | 13.8 | 10.2 | 11.7 |
| | 36:6 | 977.6 | 595.6 | 13.6 | 10.3 | 12.2 |
| | 37:4 | 995.6 | 613.6 | 13.2 | 10.7 | 11.6 |
| | 38:2 | 1013.6 | 631.6 | 13.4 | 10.1 | 11.5 |
| | 38:3 | 1011.6 | 629.6 | 13.7 | 10.1 | 11.4 |
| | 38:4 | 1009.6 | 627.6 | 13.7 | 10.6 | 11.4 |
| | 38:5 | 1007.6 | 625.6 | 13.3 | 10.4 | 11.8 |
| | 38:6 | 1005.6 | 623.6 | 14.5 | 11.9 | 12.7 |
| | 40:3 | 1039.6 | 657.6 | 15.9 | 9.8 | 11.8 |
| | 40:4 | 1037.6 | 655.6 | 13.6 | 9.7 | 11.4 |
| | 40:5 | 1035.6 | 653.6 | 14.0 | 10.0 | 12.0 |
| | 40:6 | 1033.6 | 651.6 | 15.1 | 10.5 | 12.5 |
| | 40:7 | 1031.6 | 649.6 | 15.3 | 10.5 | 12.5 |

| | Fatty acyl species | Q1 Mass (Da) | Q3 Mass (Da) | Retention Time (min) |
|---|---|---|---|---|
| | | | | PI(3,4,5)P3 |
| PIP3 | 32:0 | 1149.6 | 551.6 | 5.0 |
| | 32:1 | 1147.6 | 549.6 | 5.1 |
| | 32:2 | 1145.6 | 547.6 | 5.2 |
| | 34:0 | 1177.6 | 579.6 | 5.2 |
| | 34:1 | 1175.6 | 577.6 | 5.3 |
| | 34:2 | 1173.6 | 575.6 | 5.3 |
| | 36:0 | 1205.6 | 607.6 | 5.4 |
| | 36:1 | 1203.6 | 605.6 | 5.7 |
| | 36:2 | 1201.6 | 603.6 | 6.3 |
| | 36:3 | 1199.6 | 601.6 | 7.4 |
| | 36:4 | 1197.6 | 599.6 | 7.3 |
| | 36:5 | 1195.6 | 597.6 | 7.3 |
| | 36:6 | 1193.6 | 595.6 | 7.3 |
| | 37:4 | 1211.6 | 613.6 | 7.2 |
| | 38:2 | 1229.6 | 631.6 | 7.3 |
| | 38:3 | 1227.6 | 629.6 | 7.2 |
| | 38:4 | 1225.6 | 627.6 | 7.1 |
| | 38:5 | 1223.6 | 625.6 | 7.7 |
| | 38:6 | 1221.6 | 623.6 | 7.0 |
| | 40:3 | 1255.6 | 657.6 | 7.2 |
| | 40:4 | 1253.6 | 655.6 | 7.4 |
| | 40:5 | 1251.6 | 653.6 | 7.9 |
| | 40:6 | 1249.6 | 651.6 | 8.1 |
| | 40:7 | 1247.6 | 649.6 | 8.1 |

| | Fatty acyl species | Q1 Mass (Da) | Q3 Mass (Da) |
|---|---|---|---|
| d6-labeled PIP | 32:0 | 939.6 | 551.6 |
| | 32:1 | 937.6 | 549.6 |
| | 32:2 | 935.6 | 547.6 |
| | 34:0 | 967.6 | 579.6 |
| | 34:1 | 965.6 | 577.6 |
| | 34:2 | 963.6 | 575.6 |
| | 36:0 | 995.6 | 607.6 |
| | 36:1 | 993.6 | 605.6 |
| | 36:2 | 991.6 | 603.6 |
| | 36:3 | 989.6 | 601.6 |
| | 36:4 | 987.6 | 599.6 |
| | 36:5 | 985.6 | 597.6 |
| | 36:6 | 983.6 | 595.6 |
| | 37:4 | 995.6 | 613.6 |
| | 38:2 | 1019.6 | 631.6 |
| | 38:3 | 1017.6 | 629.6 |
| | 38:4 | 1015.6 | 627.6 |
| | 38:5 | 1013.6 | 625.6 |
| | 38:6 | 1011.6 | 623.6 |
| | 40:3 | 1045.6 | 657.6 |
| | 40:4 | 1043.6 | 655.6 |
| | 40:5 | 1041.6 | 653.6 |
| | 40:6 | 1039.6 | 651.6 |
| | 40:7 | 1037.6 | 649.6 |

| | Fatty acyl species | Q1 Mass (Da) | Q3 Mass (Da) | Retention Time (min) | | |
|---|---|---|---|---|---|---|
| | | | | PI(3,4)P2 | PI(3,5)P2 | PI(4,5)P2 |
| PIP2 | 32:0 | 1041.6 | 551.6 | 8.2 | 7.3 | 5.2 |
| | 32:1 | 1039.6 | 549.6 | 8.2 | 7.3 | 5.2 |
| | 32:2 | 1037.6 | 547.6 | 8.2 | 7.4 | 5.3 |
| | 34:0 | 1069.6 | 579.6 | 8.1 | 7.3 | 5.1 |
| | 34:1 | 1067.6 | 577.6 | 8.3 | 7.5 | 5.2 |
| | 34:2 | 1065.6 | 575.6 | 8.8 | 8.1 | 6.7 |
| | 36:0 | 1097.6 | 607.6 | 8.1 | 7.3 | 5.1 |
| | 36:1 | 1095.6 | 605.6 | 8.8 | 7.9 | 5.2 |
| | 36:2 | 1093.6 | 603.6 | 8.8 | 8.0 | 6.6 |
| | 36:3 | 1091.6 | 601.6 | 9.0 | 8.1 | 7.1 |
| | 36:4 | 1089.6 | 599.6 | 8.9 | 8.3 | 7.1 |
| | 36:5 | 1087.6 | 597.6 | 9.6 | 9.1 | 8.1 |
| | 36:6 | 1085.6 | 595.6 | 9.8 | 9.5 | 8.5 |
| | 37:4 | 1103.6 | 613.6 | 9.5 | 8.2 | 7.0 |
| | 38:2 | 1121.6 | 631.6 | 9.2 | 8.8 | 7.3 |
| | 38:3 | 1119.6 | 629.6 | 8.9 | 8.2 | 7.4 |
| | 38:4 | 1117.6 | 627.6 | 8.9 | 8.3 | 6.8 |
| | 38:5 | 1115.6 | 625.6 | 9.6 | 9.0 | 7.9 |
| | 38:6 | 1113.6 | 623.6 | 10.2 | 9.5 | 8.4 |
| | 40:3 | 1147.6 | 657.6 | 8.7 | 8.3 | 7.7 |
| | 40:4 | 1145.6 | 655.6 | 9.0 | 8.5 | 7.9 |
| | 40:5 | 1143.6 | 653.6 | 9.7 | 9.1 | 8.1 |
| | 40:6 | 1141.6 | 651.6 | 9.9 | 9.3 | 8.3 |
| | 40:7 | 1139.6 | 649.6 | 10.5 | 9.7 | 8.9 |

| | Fatty acyl species | Q1 Mass (Da) | Q3 Mass (Da) | Retention Time (min) |
|---|---|---|---|---|
| | | | | PI |
| PI | 25:0 | 727.6 | 453.6 | 16.0 |
| | 32:0 | 825.6 | 551.6 | 15.7 |
| | 32:1 | 823.6 | 549.6 | 16.6 |
| | 32:2 | 821.6 | 547.6 | 16.8 |
| | 34:0 | 853.6 | 579.6 | 15.4 |
| | 34:1 | 851.6 | 577.6 | 15.8 |
| | 34:2 | 849.6 | 575.6 | 15.1 |
| | 36:0 | 881.6 | 607.6 | 15.6 |
| | 36:1 | 879.6 | 605.6 | 17.2 |
| | 36:2 | 877.6 | 603.6 | 17.2 |
| | 36:3 | 875.6 | 601.6 | 17.2 |
| | 36:4 | 873.6 | 599.6 | 17.2 |
| | 36:5 | 871.6 | 597.6 | 17.8 |
| | 36:6 | 869.6 | 595.6 | 17.7 |
| | 38:2 | 905.6 | 631.6 | 17.1 |
| | 38:3 | 903.6 | 629.6 | 17.2 |
| | 38:4 | 901.6 | 627.6 | 17.2 |
| | 38:5 | 899.6 | 625.6 | 17.4 |
| | 38:6 | 897.6 | 623.6 | 18.0 |
| | 40:3 | 931.6 | 657.6 | 16.2 |
| | 40:4 | 929.6 | 655.6 | 17.5 |
| | 40:5 | 927.6 | 653.6 | 17.9 |
| | 40:6 | 925.6 | 651.6 | 17.9 |
| | 40:7 | 923.6 | 649.6 | 17.2 |

| | Fatty acyl species | Q1 Mass (Da) | Q3 Mass (Da) |
|---|---|---|---|
| d6-labeled PIP2 | 32:0 | 1047.6 | 551.6 |
| | 32:1 | 1045.6 | 549.6 |
| | 32:2 | 1043.6 | 547.6 |
| | 34:0 | 1075.6 | 579.6 |
| | 34:1 | 1073.6 | 577.6 |
| | 34:2 | 1071.6 | 575.6 |
| | 36:0 | 1103.6 | 607.6 |
| | 36:1 | 1101.6 | 605.6 |
| | 36:2 | 1099.6 | 603.6 |
| | 36:3 | 1097.6 | 601.6 |
| | 36:4 | 1095.6 | 599.6 |
| | 36:5 | 1093.6 | 597.6 |
| | 36:6 | 1091.6 | 595.6 |
| | 37:4 | 1103.6 | 613.6 |
| | 38:2 | 1127.6 | 631.6 |
| | 38:3 | 1125.6 | 629.6 |
| | 38:4 | 1123.6 | 627.6 |
| | 38:5 | 1121.6 | 625.6 |
| | 38:6 | 1119.6 | 623.6 |
| | 40:3 | 1153.6 | 657.6 |
| | 40:4 | 1151.6 | 655.6 |
| | 40:5 | 1149.6 | 653.6 |
| | 40:6 | 1147.6 | 651.6 |
| | 40:7 | 1145.6 | 649.6 |

| Fatty acyl species | Q1 Mass (Da) | Q3 Mass (Da) |
|---|---|---|
| 18:0/18:1-PIP | 987.6 | 341.3 |
| 18:0/18:2-PIP | 985.6 | 341.3 |
| 18:1/18:1-PIP | 985.6 | 339.3 |
| 16:0/20:2-PIP | 985.6 | 313.3 |
| 16:1/20:1-PIP | 985.6 | 311.3 |
| 18:0/20:4-PIP | 1009.6 | 341.3 |
| 18:0/18:1-PIP2 | 1095.6 | 341.3 |
| 18:0/18:2-PIP2 | 1093.6 | 341.3 |
| 18:1/18:1-PIP2 | 1093.6 | 339.3 |
| 16:0/20:2-PIP2 | 1093.6 | 313.3 |
| 16:1/20:1-PIP2 | 1093.6 | 311.3 |
| 18:0/20:4-PIP2 | 933.6 | 341.3 |

**Supplementary Table 2**. Comparison of quantitative value of PIPs between in the present study and that in the literatures

| Reference | The present study | | Zolov et al. (Ref. 11) | | Guilou et al. (Ref. 4) | |
|---|---|---|---|---|---|---|
| Cell types | MEF | | Fibroblasts | | Neutrophile | |
| Measuring method | Methylation followed by SFC-MS/MS | | Myo-[2-3H]inositol-labbeling followed by HPLC | | [32P]Pi-labbeling followed by HPLC | |
| | pmol/10^6 cells | % of total PIPs | % of total PIPs + PI | % of total PIPs | Relative propotion of PI (%) | % of total PIPs |
| PI3P | 4.6 | 9.05 | 0.17 | 2.1 | 0.84 | 0.84 |
| PI4P | 18.4 | 36.22 | 3 | 37.06 | 37.2 | 37.2 |
| PI5P | 4.1 | 8.07 | 0.29 | 3.58 | No data | - |
| PI(3,4)P2 | 1.3 | 2.55 | No data | - | 0.23 | 0.23 |
| PI(3,5)P2 | 1.9 | 3.74 | 0.035 | 0.43 | No data | - |
| PI(4,5)P2 | 20.5 | 40.35 | 4.6 | 56.82 | 61.7 | 61.7 |
| PI(3,4,5)P3 | | | No data | - | 0.03 | 0.03 |
