# Supplementary material for: Supercritical fluid chromatography-mass spectrometry enables simultaneous measurement of all phosphoinositide regioisomers
Source: Commun Chem. 2022 May 11;5:61. doi: 10.1038/s42004-022-00676-6 (PMC9814602; doi:10.1038/s42004-022-00676-6)
Supplement: Supplementary file 5 — Reporting Summary [file 42004_2022_676_MOESM5_ESM.pdf]

nature portfolio

Corresponding author(s): Nozomu Kono

Last updated by author(s): Apr 3, 2020

Reporting Summary

Nature Portfolio wishes to improve the reproducibility of the work that we publish. This form provides structure for consistency and transparency in reporting. For further information on Nature Portfolio policies, see our Editorial Policies and the Editorial Policy Checklist.

Statistics

For all statistical analyses, confirm that the following items are present in the figure legend, table legend, main text, or Methods section.

n/a	Confirmed	
☐	☒	The exact sample size (*n*) for each experimental group/condition, given as a discrete number and unit of measurement
☐	☒	A statement on whether measurements were taken from distinct samples or whether the same sample was measured repeatedly
☐	☒	The statistical test(s) used AND whether they are one- or two-sided<br>*Only common tests should be described solely by name; describe more complex techniques in the Methods section.*
☐	☒	A description of all covariates tested
☐	☒	A description of any assumptions or corrections, such as tests of normality and adjustment for multiple comparisons
☐	☒	A full description of the statistical parameters including central tendency (e.g. means) or other basic estimates (e.g. regression coefficient) AND variation (e.g. standard deviation) or associated estimates of uncertainty (e.g. confidence intervals)
☐	☒	For null hypothesis testing, the test statistic (e.g. *F*, *t*, *r*) with confidence intervals, effect sizes, degrees of freedom and *P* value noted<br>*Give P values as exact values whenever suitable.*
☒	☐	For Bayesian analysis, information on the choice of priors and Markov chain Monte Carlo settings
☐	☒	For hierarchical and complex designs, identification of the appropriate level for tests and full reporting of outcomes
☒	☐	Estimates of effect sizes (e.g. Cohen's *d*, Pearson's *r*), indicating how they were calculated

Our web collection on statistics for biologists contains articles on many of the points above.

Software and code

Policy information about availability of computer code

Data collection	1. The commercial software of masspec, Analyst (ABSciex) and Labsolutions (Shimadzu)<br>2. The commercial software of ImageQuant LAS 4000 biomolecular imager (GE Healthcare)
Data analysis	1. GraphPad Prism 8 (GraphPad)<br>2. MultiQuant (ABSciex)<br>3. Labsolutions (Shimadzu)<br>4. Shinyheatmap (http://www .shinyheatmap.com) using Euclidiean distance metrics and complete linkage algorithms (Khomtchouk et al. 2017).

For manuscripts utilizing custom algorithms or software that are central to the research but not yet described in published literature, software must be made available to editors and reviewers. We strongly encourage code deposition in a community repository (e.g. GitHub). See the Nature Portfolio guidelines for submitting code & software for further information.

Data

Policy information about availability of data

All manuscripts must include a data availability statement. This statement should provide the following information, where applicable:

- Accession codes, unique identifiers, or web links for publicly available datasets
- A description of any restrictions on data availability
- For clinical datasets or third party data, please ensure that the statement adheres to our policy

The datasets generated during and/or analyzed during the current study are available in the manuscript, the Supplementary Information or the Supplementary Data and are available from the corresponding author upon request.

March 2021

Field-specific reporting

Please select the one below that is the best fit for your research. If you are not sure, read the appropriate sections before making your selection.

☒ Life sciences ☐ Behavioural & social sciences ☐ Ecological, evolutionary & environmental sciences

For a reference copy of the document with all sections, see nature.com/documents/nr-reporting-summary-flat.pdf

Life sciences study design

All studies must disclose on these points even when the disclosure is negative.

Sample size	No statistical methods were used to predetermine sample size. Samples sizes were chosen based on previous experience in our laboratory.
Data exclusions	No data were excluded from the analysis.
Replication	All attempts at replication were successful.
Randomization	Cells and animals were not randomly allocated into experimental groups.
Blinding	The experiments were performed and analyzed in non-blinded fashions. The experimental conditions were obvious to the researchers and the analyses were performed objectively and not subjective to human bias.

Reporting for specific materials, systems and methods

We require information from authors about some types of materials, experimental systems and methods used in many studies. Here, indicate whether each material, system or method listed is relevant to your study. If you are not sure if a list item applies to your research, read the appropriate section before selecting a response.

Materials & experimental systems

n/a	Involved in the study	
☐	☒	Antibodies
☐	☒	Eukaryotic cell lines
☒	☐	Palaeontology and archaeology
☐	☒	Animals and other organisms
☒	☐	Human research participants
☒	☐	Clinical data
☒	☐	Dual use research of concern

Methods

n/a	Involved in the study	
☒	☐	ChIP-seq
☒	☐	Flow cytometry
☒	☐	MRI-based neuroimaging

Antibodies

Antibodies used	Mouse monoclonal anti-GAPDH (6C5; Calbiochem)<br>Rat monoclonal anti-LPIAT1 antibody (clone: FT10) was produced in our laboratory.<br>Mouse monoclonal anti-VPS34 (sc-365404; Santa Cruz)<br>Mouse monoclonal anti-Pikfyve (sc-100408; Santa Cruz)<br>Rabbit polyclonal anti-PI4Kα (12411-1-AP; Proteintech)<br>Mouse monoclonal anti-PI4Kβ (611816; BD laboratory)
Validation	All antibodies were validated either by the manufacturer or used extensively in published research article.

Eukaryotic cell lines

Policy information about cell lines

Cell line source(s)	FIP200 KO MEFs and control MEFs were generated previously in Jun-Lin Guan Lab (Gan, B. et al. J. Cell Biol. 175, 121–133 (2006).)
Authentication	The cell lines were authenticated based on the morphology under microscope and the analysis of the growth curve.
Mycoplasma contamination	All cells are routinely tested for mycoplasma contamination.
Commonly misidentified lines (See ICLAC register)	Not applicable.

Animals and other organisms

Policy information about studies involving animals; ARRIVE guidelines recommended for reporting animal research

Laboratory animals	Wild-type mice (C57BL/6J) were purchased from CLEA Japan. All mice were housed in climate-controlled (23 °C) specific pathogen-free facilities with a 12-h light-dark cycle, with free access to standard laboratory food (CE2; CLEA) and water.
Wild animals	Not applicable.
Field-collected samples	Not applicable.
Ethics oversight	All animal experiments were performed in accordance with protocols approved by Animal Committees of the University of Tokyo in accordance with the Standards Relating to the Care and Management of Experimental Animals in Japan.

Note that full information on the approval of the study protocol must also be provided in the manuscript.
