# Supplementary material for: Supercritical fluid chromatography-mass spectrometry enables simultaneous measurement of all phosphoinositide regioisomers
Source: Commun Chem. 2022 May 11;5:61. doi: 10.1038/s42004-022-00676-6 (PMC9814602; doi:10.1038/s42004-022-00676-6)
Supplement: Supplementary file 4 — Peer Review File [file 42004_2022_676_MOESM4_ESM.pdf]

nature portfolio

Peer Review File

Supercritical fluid chromatography-mass spectrometry enables simultaneous measurement of all phosphoinositide regioisomers

Open Access This file is licensed under a Creative Commons Attribution 4.0 International License, which permits use, sharing, adaptation, distribution and reproduction in any medium or format, as long as you give appropriate credit to the original author(s) and the source, provide a link to the Creative Commons license, and indicate if changes were made. In the cases where the authors are anonymous, such as is the case for the reports of anonymous peer reviewers, author attribution should be to 'Anonymous Referee' followed by a clear attribution to the source work. The images or other third party material in this file are included in the article's Creative Commons license, unless indicated otherwise in a credit line to the material. If material is not included in the article's Creative Commons license and your intended use is not permitted by statutory regulation or exceeds the permitted use, you will need to obtain permission directly from the copyright holder. To view a copy of this license, visit http://creativecommons.org/licenses/by/4.0/.

This manuscript has been previously reviewed at another Nature Portfolio journal. This document only contains reviewer comments and rebuttal letters for versions considered at Communications Chemistry.

REVIEWERS' COMMENTS:

Reviewer #1 (Remarks to the Author):

The authors have adequately answered the points raised by the reviewer. Thank you for improving the study which appears now suitable for publication.

Reviewer #2 (Remarks to the Author):

Previous critiques have been properly addressed by the investigators. The quality of the manuscript improved significantly and is ready for publication.
